# Apelin-13 confers Neuropeptide Y–mediated neuroprotection and preserves learning and allocentric memory in D-glutamic acid-induced excitotoxicity in rats

**DOI:** 10.1007/s12035-026-05685-3

**Published:** 2026-01-22

**Authors:** Kadriye Yagmur Oruc, Aykut Oruc, Ruhat Arslan, Furkan Pasa Diriarin, Murat Mengi, Gamze Tanriverdi, Karolin Yanar, Mediha Ozeren Eser, Gokhan Agturk, Ali Ihsan Sonkurt, Berkay Guler, Hakki Oktay Seymen

**Affiliations:** 1 Physiology / Cerrahpasa Faculty of Medicine, Istanbul University-Cerrahpaşa, Istanbul, Turkey; 2 Physiology/ Faculty of Medicine, Istinye University, Istanbul, Turkey; 3 Physiology / Faculty of Medicine, Tekirdağ Namık Kemal University, Tekirdag, Turkey; 4 Histology And Embryology / Cerrahpasa Faculty of Medicine, Istanbul University-Cerrahpaşa, Istanbul, Turkey; 5 Medical Biochemistry, Cerrahpasa Faculty of Medicine, Istanbul University-Cerrahpaşa, Istanbul, Turkey; 6 Histology And Embryology / Faculty of Medicine, Altınbaş University, Istanbul, Turkey; 7 Physiology / Faculty of Medicine, Haliç University, Istanbul, Turkey; 8 Medical School, Cerrahpasa Faculty of Medicine, Istanbul University-Cerrahpaşa, Istanbul, Turkey

**Keywords:** Excitotoxicity, Apelin-13, Neuropeptide Y, Allocentric Memory, Spatial Memory

## Abstract

Glutamate-mediated excitotoxicity leads to mitochondrial dysfunction, apoptosis, and neuronal cell death. This study aims to investigate whether NPY2 receptors (NPY2R) and NPY5 receptors (NPY5R) enhance the effects of Apelin-13/APJ signaling pathways as modulatory cofactors in the neuroprotection provided by Apelin-13 against excitotoxic damage and in the prevention of learning-memory disorders. D-Glutamic acid-induced excitotoxicity was established in 42 male Sprague–Dawley rats (6–8 weeks, 200-250 g). Animals were randomly divided into six groups (n = 7); Control (C; 0.9% NaCl, i.p), D-Glutamic Acid (G; 4 mg/kg, i.p), Apelin-13 (A; 300 µg/kg, i.p), D-Glutamic Acid + Apelin-13 (GA), D-Glutamic Acid + Apelin-13 + NPY2R antagonist (GAN2; 1,5 mg/kg, i.p) and D-Glutamic Acid + Apelin-13 + NPY5R antagonist (GAN5; 1,5 mg/kg, i.p). Locomotor activity were evaluated with the Open Field (OFT), short/long-term memory and learning performance, allocentric-egocentric orientation were assesed with novel object recognition (NORT) and Morris water maze (MWM) tests. All parameters were normalized to the C group, and statistical significance between groups was assesed. In group G, a significant decrease (p < 0.001) in Extracellular Signal Regulatory Kinase (ERK1/2) and Protein Kinase B-1 (AKT-1) levels and an increase (p < 0.001) in Caspase-3 were observed. Oxidative parameters increased in the G and GAN2 groups. Antioxidant parameters were also elevated in GA and GAN5, similar to C and A groups. An increase in MWM latency to the target quadrant (p < 0.001) and a decrease in NORT discrimination index (p < 0.001) were found in the G and GAN2 groups compare to the C and A. Histochemical staining scores showed that the protection of Apelin-13 was mediated by NPY2R. In GAN2, blocking NPY2R reduces Apelin-13’s neuroprotection, which is sustained only via NPY5R with limited effect. In GAN5, Apelin-13’s protection was enhanced through NPY2R, as shown with NPY5R blockade. Accordingly, Apelin-13 exerts its neuroprotective effects primarily through NPY2R, its modulatory influence via NPY5R appears to be comparatively limited.

**Graphical Abstract:**

Graphical abstract illustrating the interplay between Apelin-13, NPY2 receptor (NPY2R) antagonist and NPY5 receptor (NPY5R) antagonist in modulating D-Glutamic acid induced excitotoxicity. **Left side:** This represents the normal glutamate excitotoxicity condition. Excessive glutamate release stimulates NMDA and AMPA receptors found in the postsynaptic membrane, leading to Na^+^ and Ca^+2^ overload, ROS/RNS production, mitochondrial dysfunction, apoptosis, neuronal death, and cognitive impairment. **Middle:** Exogenous administration of Apelin-13 along with an NPY2R antagonist results in selective blockade of NPY2R while leaving NPY5R active. NPY2R blockade fails to inhibit presynaptic glutamate release, allowing continued activation of NMDA and AMPA receptors. Therefore, the neuroprotective effects of Apelin-13 are confined to NPY5R-mediated pathways. **Right:** When Apelin-13 is administered together with the NPY5R antagonist results in selective blockade of NPY5R while leaving NPY2R active. NPY2R activation inhibits presynaptic glutamate release, Apelin-13 also inhibits NMDA and AMPA receptors. Consequently, the neuroprotective effects of Apelin-13 are predominantly dependent on NPY2R.

VGCC: Voltage-gated calcium channel; Glu: Glutamate; APJ: Apelin receptor; NPY2R/NPY5R: Neuropeptide Y receptors 2 and 5; NPY: Neuropeptide Y; ROS/RNS: Reactive oxygen/nitrogen species, AMPA: α-amino-3-hydroxy-5-methyl-4-isoxazolepropionic acid; NMDA: N-methyl-D-aspartate; Ca^+2^: Calcium, Na^+^: Sodium.

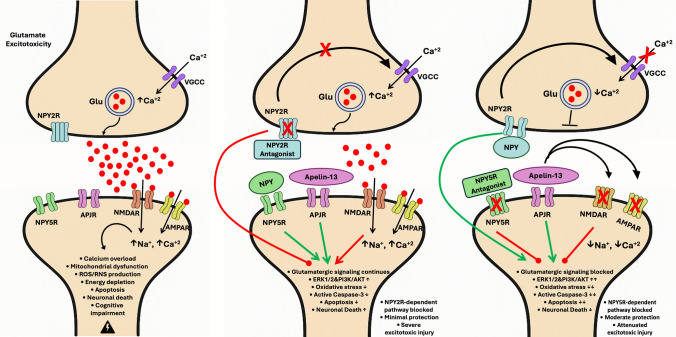

## Introductıon

Glutamate, the primary excitatory neurotransmitter in the central nervous system (CNS), plays a crucial role in neuronal communication, synaptogenesis, and learning and memory processes through its ionotropic (iGluR) and metabotropic receptors (mGluR) [1]. Dysregulation of the glutamatergic system can trigger neurotoxic events at the cellular level, such as oxidative stress excitotoxicity, and mitochondrial dysfunction, leading to many neurological pathologies such as Alzheimer's Disease (AD), Parkinson's Disease (PD), and epilepsy [1–7]. Excitotoxicity is a pathological mechanism that can develop acutely due to sudden calcium discharge resulting from excessive glutamate stimulation of postsynaptic neurons, or chronically due to sustained elevations in synaptic basal glutamate concentration above the physiological levels, leading to neuronal death [8]. The basal concentration of glutamate in the synaptic cleft varies between 25 and 600 nM [9, 10], and this value is insufficient to activate glutamate receptors [8–11]. However, sustained elevations in basal glutamate concentrations, reaching or exceeding 2–5 μM in the synaptic cleft, can cause severe excitotoxic damage [8, 10]. This condition involves NMDAR/AMPAR overactivation, calcium (Ca^2^⁺) dysregulation, and impairment of the glutamate-glutamine cycle, leading to reactive oxygen species (ROS) accumulation, oxidative stress, mitochondrial dysfunction, and apoptosis [8, 12, 13]. Collectively, this molecular damage leads to synaptic loss at the neural circuit level, neuromorphological abnormalities, disruptions in learning and memory processes, and cognitive-behavioral impairments [8].

Endogenous molecules with neuroprotective effects, such as Apelin-13, attract attention in the regulation of pathways involved in the pathogenesis of excitotoxicity. Apelin, a neuropeptide in the central nervous system, is an endogenous adipokine produced from adipose tissue [14, 15]. It is the ligand of the Apelin receptor (APJ), a G protein-coupled receptor (GPCR). Initially produced as a 77-amino-acid pre-proapelin, apelin is degraded by endogenous peptidases to its active isoforms, such as 13, 17, and 36 [15–18]. Apelin-13 is the most potent and predominant isoform in plasma and can cross the blood–brain barrier via peripheral pathways [19–21]. Apelin-13/APJ complex regulates intracellular Ca^2+^ by inhibiting NMDAR/AMPAR via enhanced extracellular signal-regulated kinase (ERK1/2) and protein kinase B (AKT) signaling. By increasing ERK1/2 and AKT signaling pathways, Apelin-13 suppresses mitochondrial dysfunction, apoptosis and oxidative stress, alleviates learning and memory impairment [9, 22, 23].

Neuropeptide Y (NPY), another endogenous neuropeptide found in the central nervous system, supports neuronal survival by increasing neurotrophic factors, provides excitotoxicity inhibition, regulates neuroprotection, Ca^2+^ homeostasis and learning-memory processes. NPY performs these effects through GPCR-mediated NPY1, -Y2, ​​​​-Y4, -Y5, -y6 receptor subtypes [24–28]. Rather than examining the general effects of NPY, the study focuses on the NPY2R and NPY5R receptor subtypes, which play a crucial role in regulating excitotoxicity. The NPY2 receptor (NPY2R) subtype is localized in the presynaptic terminals of the hippocampus, including the dentate gyrus (DG), Cornu Ammonis 1 (CA1), CA3 areas, amygdala, thalamus, hypothalamus, and cerebral cortex. The NPY5 receptor (NPY5R) subtype is localized postsynaptically in the hippocampus DG, CA1, CA2, CA3, cingulate cortex, and hypothalamic nuclei [28–30]. NPY prevents excitotoxic damage by regulating glutamate release from the CA1 presynaptic terminal via NPY2R and postsynaptic NPY5R in CA1-CA3 with ERK1/2, PI3K/AKT signaling [31–37]. Changes in NPY levels have been observed in neurodegenerative diseases such as AD, PD [27, 28, 38]. Additionally, NPY2R enhances consolidation in spatial memory [39].

The hippocampus, a critical structure involved in the learning and memory process, plays a crucial role in spatial navigation [40]. Allocentric and egocentric navigation are accepted coding systems in spatial mapping. In allocentric coding, the position of an object is evaluated by reference to the positions of other objects. In contrast, in egocentric coding, the position of objects in space is represented according to the individual's body axes, that is, the reference point is the individual himself [41]. In spatial mapping, hippocampus-dependent allocentric and hippocampus-independent egocentric strategies gain importance in neurodegenerative disease progression. In hippocampal damage, spatial mapping is disrupted, and allocentric navigation is decreased. Prolonged search time and distance characterize a compensatory shift toward egocentric strategies [40]. Mitochondrial dysfunction, oxidative stress, and misfolded protein accumulation lead to excitotoxicity, causing learning and memory impairment in hippocampal circuits [11, 42]. The prefrontal cortex (PFC) is associated with cognitive performance and short-term memory [43, 44]. The PFC plays a key role in organizing information in short-term memory (STM) and transferring it to long-term memory (LTM) [45]. The sensitivity of PFC neurons to glutamatergic excitotoxicity leads to neuronal dysfunction and the impairment of short-term memory [43].

Oxidative and nitrative injuries play a mechanistic role in the pathology of neurodegenerative diseases [46]. Mitochondrial dysfunction, protein aggregation, and decreased cellular antioxidant defense mechanisms lead to increased oxidative stress in neuronal cells, resulting in lipid peroxidation [47]. Advanced Oxidation Protein Products (AOPP), Advanced Glycation End-products (AGE) indicates protein peroxidation and glycoxidation, while Malondialdehyde (MDA) levels, which indicate the level of lipid peroxidation, are increased in neurodegenerative diseases [48–51]. Kaynurenine (KYN) and dityrosine (DT) levels, which indicate nitrosative stress, increase in neurodegeneration [52, 53]. Total thiol (T‑SH) levels, which serve as general indicators of antioxidant capacity, have been shown to decrease in excitotoxic damage [54].

Although the neuroprotective effects of apelin-13 have been demonstrated, the multifactorial nature of the pathophysiological mechanisms makes the specificity of this effect unclear. The colocalization of APJ with NPY2R and NPY5R in hippocampal areas sensitive to excitotoxic damage, along with their similar protective functions via ERK1/2 and PI3K/AKT signaling pathways, has highlighted the idea that Apelin-13 and NPY work in coordination against excitotoxicity [18, 55, 56].

To induce excitotoxic neuronal damage, we used D‑glutamic acid, which accumulates in tissues due to its resistance to deamination and crosses the blood–brain barrier [57–59]. Its metabolism by stereospecific oxidases such as D‑aspartate oxidase generates hydrogen peroxide, a reactive oxygen species that impairs cellular integrity and function by promoting oxidative stress [12, 60, 61]. Hydrogen peroxide contributes to mitochondrial dysfunction, neuroinflammation, and cell death by inducing apoptosis through upregulation of pro‑apoptotic genes (tumor protein 53 (tp53), caspase 3 (casp3), BCL2 associated X protein (bax)) and downregulation of anti‑apoptotic B-cell lymphoma 2 alpha isoform (bcl2α). It also suppresses the antioxidant defense system by reducing superoxide dismutase 1 (sod1), catalase (cat), and glutathione peroxidase 1a (gpx1a) gene expression, further exacerbating neuronal vulnerability [62, 63]. Therefore, D‑glutamic acid was chosen not only as an excitotoxic agent but also as an inducer of oxidative stress, reflecting key pathological mechanisms involved in neurodegeneration. Compared to the commonly used L-glutamic acid, L-glutamate, or monosodium glutamate-based models to induce excitotoxicity in the literature, studies using D-glutamic acid are quite limited [60, 64–66].

To date, no studies have examined explicitly whether Apelin-13 and NPY neuropeptides act in concert to counteract excitotoxic damage. In this study, we aimed to investigate the neuroprotective and learning and memory effects of Apelin-13 in the D-glutamic acid-induced excitotoxicity model and the relationship between NPY2R and NPY5R.

## Method

### Chemicals

D-glutamic acid (D-Glutamic acid, Sigma, CAS:6893–26-1, Saint Louis, USA), Apelin-13 (Apelin-13, Cayman Chemicals, Item 13,523, Michigan, USA), NPY Y2 receptor antagonist (BIIE 0246 hydrochloride, cat no 7377, Tocris Bioscience, Bristol, UK), NPY Y5 receptor antagonist (L-152.804, catalog no 1382, Tocris Bioscience, Bristol, UK), Ketamine Hydrochloride (HCl) (Ketalar HCl 50 mg/mL, Pfizer, Istanbul, Turkey), Xylazine HCl (Rompun 20 mg/mL, Bayer, Istanbul, Turkey), Dimethylsulfoxide (Dimethyl Sulfoxide EMPLURA, Cat no 116743, Merck, Darmstadt, Germany) were used while performing the experiments.

### Animals

Forty-two male Sprague–Dawley rats, 6–8 weeks old, weighing 200–250 g, belonging to the same generation, were obtained from the Istanbul University-Cerrahpaşa Nanotechnology and Biotechnology Institute Experimental Medicine Research Laboratory (DETALAB). Ethical approval was obtained from Istanbul University-Cerrahpaşa Animal Experiments Local Ethics Committee (approval number: 2023/11) (Clinical trial number: not applicable.). The animals were housed in a 12-h light/dark cycle, with 55 ± 10% humidity and 23 ± 1 °C. They were fed with standard pellet food and water ad libitum. The cages were taken into the experimental room 1 week before the experiment and cleaned every other day. Rats were randomly assigned to six groups (n = 7, per group): Control (C), Apelin-13 (A), D-Glutamic acid (G), D-Glutamic acid + Apelin-13 (GA), D-Glutamic acid + NPY 2 Receptor Antagonist + Apelin-13 (GAN2), D-Glutamic acid + NPY 5 Receptor Antagonist + Apelin-13 (GAN5).

- A vehicle stock solution was prepared by mixing 1 mL of dimethyl sulfoxide (DMSO) with 0.9% NaCl (physiological saline, PS).
- D-glutamic acid was dissolved in 1 mL of DMSO and completed with 0.9% NaCl physiological serum to prepare a 4 mg/kg D-glutamic acid stock solution.
- Apelin-13 was dissolved in 1 mL of DMSO and completed with 0.9% NaCl physiological serum to prepare a 300 µg/kg Apelin-13 stock solution.
- The NPY2R antagonist was dissolved in 1 mL of DMSO and completed with 0.9% NaCl physiological serum to prepare a 1.5 mg/kg NPY2R antagonist stock solution.
- The NPY5R antagonist was dissolved in 1 mL of DMSO and completed with 0.9% NaCl physiological serum to prepare a 1.5 mg/kg NPY5R antagonist stock solution. Final DMSO concentration was 0.8% (v/v) across all groups.

For the establishment of the D-glutamic acid-mediated excitotoxicity model, the G, GA, GAN2, and GAN5 groups received a single injection of D-glutamic acid via the i.p. route on the 1st day of the experiment. The 4 mg/kg dose of D-glutamic acid was determined based on both previous MSG‑based excitotoxicity models, which used comparable doses to induce neuronal damage with minimal mortality [67, 68], and on preliminary experiments conducted in our laboratory to optimize efficacy and safety [60, 61]. D‑glutamic acid and MSG share similar glutamate‑mediated mechanisms of excitotoxicity, but D‑glutamic acid was preferred here as a more defined and stereospecific model. Since the C and A groups were not damage groups, they received i.p. injections from the vehicle stock solution for standardization purposes on the first day of the experiment. All groups waited 3 days for the establishment of the model, during which they did not receive any injections; they were fed ad libitum. Injections were initiated 72 h after D‑glutamic acid administration, allowing sufficient time for excitotoxic hippocampal damage and associated inflammatory processes to stabilize. This timing is consistent with previous studies showing that excitotoxic and ischemic injury becomes prominent and measurable within 48–72 h [60, 69, 70].

**Fig. 1:**
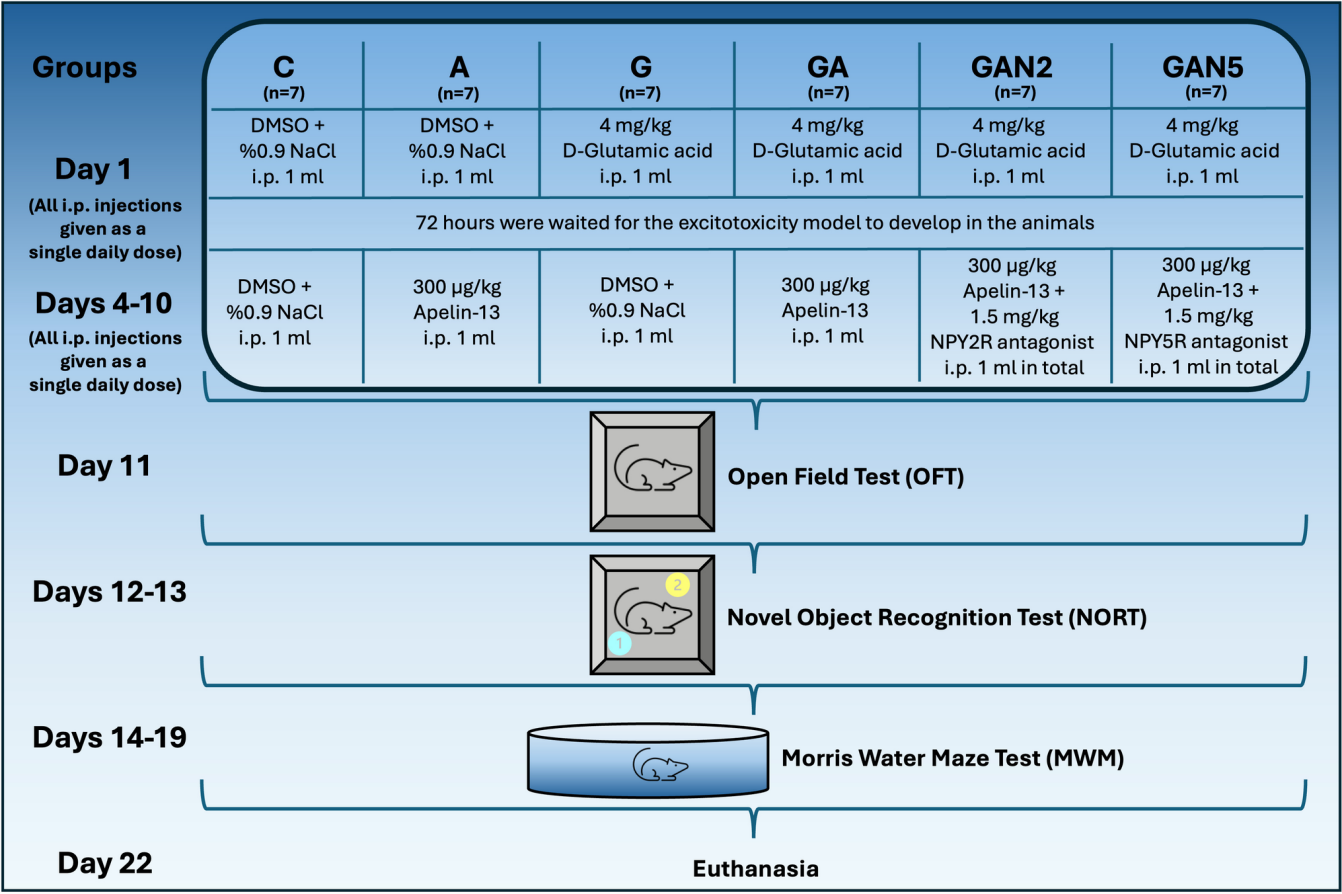
Experimental design and timeline of the study. Rats were randomly divided into six groups (*n* = 7, per group). Control (C), Apelin-13 (A), D-Glutamic acid (G), D-Glutamic acit + Apelin-13 (GA), D-glutamic acid + Apelin-13 + NPY2R antagonist (GAN2), and D-glutamic acid + Apelin-13 + NPY5R antagonist (GAN5). On Day 1, animals received a single dose of intraperitoneal (i.p.) injection of their assigned treatment. On Days 4–10, daily i.p. injections were administered as per group allocation. 72-h period was allowed for the excitotoxicity model to develop after D-glutamic acid administration. Behavioral assessments included the Open Field Test (OFT) (Day 11), Novel Object Recognition Test (NORT) (Days 12–13), and Morris Water Maze (Days 14–19). Animals were euthanized on Day 22

- Group C (n = 7) received a 1 mL volume of vehicle stock solution as a single daily dose via the i.p. route on the 1st day of the experiment. From days 4 to 10, a vehicle stock solution was administered as a single daily dose of 1 ml via i.p. injection.
- Group A (n = 7) received a 1 mL volume of vehicle stock solution as a single daily dose via the i.p. route on the 1st day of the experiment. From days 4 to 10, a single daily dose of 300 µg/kg Apelin-13 [71–74], stock solution was administered via intraperitoneal (i.p.) injection in a volume of 1 mL.
- Group G (n = 7) received a 1 mL volume of a 4 mg/kg D-glutamic acid stock solution, administered as a single daily dose via the i.p. route on the 1st day of the experiment. From days 4 to 10, a vehicle stock solution was administered as a single daily dose of 1 ml via i.p. injection.
- Group GA (n = 7) received a 1 mL volume of a 4 mg/kg D-glutamic acid stock solution as a single daily dose via the i.p. route on the 1st day of the experiment. From days 4 to 10, a single daily dose of 300 µg/kg Apelin-13 stock solution was administered via i.p. injection in a volume of 1 ml.
- Group GAN2 (n = 7) received a 1 mL volume of a 4 mg/kg D-glutamic acid stock solution, administered as a single daily dose via the i.p. route on the 1st day of the experiment. From days 4 to 10, a 300 µg/kg Apelin-13 stock solution and a NPY2R antagonist stock solution were administered as a single daily dose in a total volume of 1 ml via i.p. injection. For the NPY2 receptor antagonist (BIIE0246), we used a dose of 1.5 mg/kg i.p., which has been widely used in the literature and demonstrated to effectively block Y₂ receptor signaling in rodent behavioral studies [75, 76].
- Group GAN5 (n = 7) received a 1 mL volume of a 4 mg/kg D-glutamic acid stock solution, administered as a single daily dose via the i.p. route on the 1st day of the experiment. From days 4 to 10, 300 µg/kg Apelin-13 stock solution and NPY5R antagonist stock solution were administered as a cocktail single daily dose in a total volume of 1 ml via i.p. injection. (Fig. 1). For the NPY5 receptor antagonist (L‑152,804), there is no prior in vivo excitotoxicity study available in the literature. However, a previous study in the context of alcohol dependence employed doses of 3–10 mg/kg i.p., identifying 3 mg/kg as the lowest effective dose [77]. Considering that alcohol models may require higher doses due to their focus on reward circuits and feeding behaviors, and to maintain consistency, comparability and to avoid potential ceiling effects with the NPY2 antagonist dose, we selected 1.5 mg/kg i.p. for L‑152,804 in our excitotoxicity model.

### Open Field Test

A 70 × 70 cm opaque, gray-colored, open-topped cardboard box was used in a room illuminated with constant light in open field test (OFT). Tests were evaluated with ANY-Maze Version 7.42 software (AnyMaze, Stoelting Co., Illinois, USA). The experiment were recorded using a Canon PowerShot SX740 HS camera (Tokyo, Japan).

Rats were placed in the center of the test box and allowed to move freely for 10 min. In the recorded videos, the average speed, total distance, number of rearings, number of defecations, time spent in the center, and time spent on the edge were evaluated. [78].

### Learning and Memory Tests

The Novel Object Recognition Test (NORT) was used to assess short-term memory and the Morris-Water Maze Test (MWM) was used to assess long-term memory. Tests were evaluated with ANY-Maze Version 7.42 software and experiments were recorded using the Canon PowerShot SX740 HS camera.

#### Novel Object Recognition Test

For NORT, a second identical box with the same properties as the box used in OFT was used. To evaluate short-term memory (STM), rats were given 5 min to explore their surroundings during the habituation period. In familiarization, identical green objects (A1, A2), measuring 11 cm × 7 cm (length x diameter), were placed diagonally in the box. The rat was placed in the middle of the objects, and contact was expected to last for at least 20 s for 10 min. After 60 min, a new object (B) measuring 11 cm was placed in place of the old A2. The rat touching the object with its nose was considered “contact”. Discrimination index (DI), average speed and distance, total examination time, and number of A1-A2 and A1-B were evaluated [79, 80].

#### Morris Water Maze Test

MWM was applied to evaluate spatial memory and long-term memory (LTM). The pool, consisting of a white Plexiglas pool (150 × 60 cm, diameter x depth) and a transparent Plexiglas platform (10 × 28 cm, diameter x length), was filled to 30 cm, painted black with non-toxic paint, and heated to 26 ± 1 °C. Blue-green shapes were placed on the walls for allocentric memory, and black shapes were placed on the pool perimeter for egocentric memory. Rats were released into the pool from different points in four consecutive trials over five days. Each trial lasted 60 s, and the inter-trial interval was set at 15 s. [81]. Probe trial was performed 24 h after the last training [81, 82]. The escape platform was removed on the probe day, and the rats were released into the pool from the northeast, the only direction from which they were not thrown, and swam for 60 s. The time to reach the target quadrant, speed, latency, average swimming speed, and distance were evaluated [81].

### Euthanasia

Before anesthesia, rats were weighed and given a cocktail of ketamine 50 mg/kg and xylazine HCl 10 mg/kg via the i.p. route [83, 84]. The ketamine/xylazine cocktail used in euthanasia does not affect the oxidative parameters statistically [85, 86]. Since euthanasia was provided with the same method in our control and experimental groups, we report that the differences between the groups are due to the experimental interventions. Following the loss of reflexes, intracardiac blood was collected. Serum samples were labeled and stored at −20 °C. Cervical dislocation was performed in accordance with ethical guidelines. The brain was sagittally dissected, and the bilateral entire hippocampus and prefrontal cortex (PFC) were removed according to the Paxinos and Watson stereotaxic atlas for standardization. The hippocampus was isolated by gently removing it from the overlying caudate-putamen nucleus region of the basal ganglia. Bregma levels for the bilateral hippocampus range from −2.00 mm to −7.00 mm horizontally and coronally, whereas prefrontal cortex ranges from −2.00 mm to + 5.00 mm horizontally and coronally. Half of the hippocampus and PFC were stored at −20 °C [87, 88] for short-term storage (analyzed within 24 h) for biochemical analyses, while the remainder was stored in 10% neutral buffered formalin [89] for histological analyses.

### Biochemical Analyses

Conducted at Istanbul University-Cerrahpasa, Cerrahpasa Medical Faculty Biochemistry Research Laboratory. MDA, AOPP, T-SH, DT, KYN, AGE, and total protein levels were analyzed in plasma, hippocampus, and PFC. The brain tissue was rinsed with phosphate-buffered saline (PBS, pH 7.4) at 4 °C, weighed, and homogenized with PBS at a ratio of 1:9. The homogenates were centrifuged, and the supernatants were labeled and stored.

#### Advanced Glycation End Products, Dityrosine, and Kynurenine

According to the procedure in the literature, 150 μL of the sample was diluted 1:50 with PBS. It was read in a spectrofluorometer at excitation/emission wavelengths of 325/440 nm for AGE, 330/415 nm for DT, and 365/480 nm for KYN [90].

#### Advanced Oxidation Protein Products

20 μL of sample and 200 μL of citric acid were added to the microplate, and the mixture was incubated for 2 min. Then, 10 μL of potassium iodide was pipetted. The samples were measured spectrophotometrically at a wavelength of 340 nm, and the AOPP concentration was determined according to the procedure in the literature [91].

#### Malondialdehyde

According to the procedure outlined in the literature [92], 2 mL of the reagent, prepared with 15% TCA, 0.375% TBA, and 0.25 mol/L HCl, was added to 1 mL of the sample and then incubated in a water bath at 95 °C for 15 min. The formed precipitate was centrifuged at 1000 RPM for 10 min. The absorbance of the supernatant was measured at 532 nm in a spectrophotometer and the MDA concentration was calculated using the molar extinction coefficient of 1.56 × 105 M-1 cm-1.

#### Total Thiol

According to the method described in the literature [93], 20 μL of sample, 400 μL of Tris buffer (pH 8.2), and 20 μL of dithionitrobenzoic acid (5,5-dithio-bis-(2-nitrobenzoic acid), Cat. No. 22582, ThermoFisher Scientific, USA) were added to the microplate. The absorbance values of the samples were analyzed against the reagent blank in a spectrophotometer at 412 nm, and the extinction coefficient and T-SH concentrations were calculated.

#### Total Protein

To determine the total protein amount in tissue and plasma, 194 μL of bicinchoninic acid (BCA) was diluted 1:50 with 4% copper sulfate. 194 μL of BCA and 6 μL of sample were pipetted. Standards were prepared with albumin and added to standard wells. Phosphate buffer was added to blank wells and incubated in the dark for 30 min. The absorbance value was measured using the spectrophotometric method at a wavelength of 562 nm. A calibration curve was created for the standards and samples to facilitate the calculation of results [94].

### Enzyme-Linked ImmunoSorbent Assay

Experiments were conducted in Istanbul University-Cerrahpasa, Cerrahpasa Faculty of Medicine Biochemistry Research Laboratory, and Haliç University Faculty of Medicine Physiology Research Laboratory. Rat extracellular regulated protein kinases 1;2 (ERK1;2) ELISA Kit (Cat no: E1479Ra, BT LAB, China), Rat RAC-alpha serine; threonine-protein kinase (AKT1) ELISA Kit (Cat no: E2548Ra, BT LAB, China) and Rat CASP3 (Caspase 3) ELISA Kit (Cat no: ELK1528, ELK Biotechnology, Denver, USA) kits were purchased from the manufacturers. All ELISA assays were performed according to the manufacturers’ instructions.

### Histological Analyses

Hematoxylin&Eosin (H&E) staining and Cresyl violet staining were performed to evaluate tissue histology in all groups. H&E staining was used to evaluate the morphological features of the hippocampal CA1-CA3 regions, specifically within the molecular layer (ML), pyramidal cell layer (PML), and polymorphic layer (PL), focusing on healthy neurons (h), pericellular halo (ph), pyknotic nuclei (pn), vacuolization (v), and apoptotic bodies (ab). Cresyl violet staining (Nissl staining) was used to assess the morphological features of the prefrontal cortex (PFC) layers—molecular (I), external granular (II), external pyramidal (III), internal granular (IV), internal pyramidal (V), and polymorphic layer (VI)—including healthy neurons (h), pericellular halo (ph), pyknotic nuclei (pn), vacuolization (v), and apoptotic bodies (ab). Tissue embedding, sectioning, and staining were performed in the Histology Research Laboratory of the Faculty of Medicine of Istanbul Altınbaş University, and histological examinations were performed in the Histology Research Laboratory of the Faculty of Medicine of Istanbul University-Cerrahpaşa. All examinations were performed in the hippocampus CA1-CA3 and PFC. PFC and hippocampus were fixed in 10% neutral formalin (4% formaldehyde, pH: 7). The tissue was dehydrated in an ascending ethanol series (from 70 to 100%) after tracking. Alcohol clearance was performed in toluene. Tissues were embedded in soft and hard paraffin for 45 min each. The hippocampus was embedded in paraffin with the subiculum oriented medially and the convex surface of the dentate gyrus facing the CA1–CA3 regions. This positioning allowed the dentate gyrus and CA fields to be visualized in opposition within the same plane in coronal sections. Considering the natural craniocaudal axis of the hippocampus, coronal sections of 5 µm thickness were obtained with a microtome (Leica RM 2125 RST, Wetzlar, Germany), and mounted onto glass slides. Deparaffinization was performed, and the samples were then rehydrated in a descending ethanol series (from 100 to 70%) for subsequent staining. Sections stained with Hematoxylin&Eosin (H&E, ABCAM, ab245880, UK) and Cresyl Violet were examined morphologically under 10x, 20 × and 40 × magnification with a light microscope (Olympus BX61, Japan) integrated camera (Olympus DP72, Japan). By modifying the literature method [95] five randomly selected fields from the 40x-magnified sections were scored blindly by two researchers. Neurons were histologically evaluated on a scale of 0–5 according to their morphological features (0: undamaged, 5: severely damaged) and statistical analysis was performed.

### Statistical Analysis

Statistical analyses were performed using GraphPad Prism (v10.2.0, Boston, MA, USA). Normality and homogeneity were assessed using the Shapiro–Wilk and Levene tests. Since the data were found to be normal and homogeneous, One-way ANOVA and post hoc Tukey test were used in ELISA, OFT, NORT Familiarization and Test day pairwise comparisons, MWM Probe and biochemical analyses. Morris water maze training was analyzed using two-way repeated measures ANOVA with Bonferroni post hoc test; NORT Familiarization and Test Day object comparisons with two-way ANOVA and Sidak post hoc test. Results are presented as mean ± standard deviation (SD). Histological scoring analyses were performed using Kruskal–Wallis test and Dunn’s multiple comparisons. Histological results were given as median with interquartile range [IQR]. p < 0.05 was considered statistically significant.

## Results

### Open Field Test

**Fig. 2:**
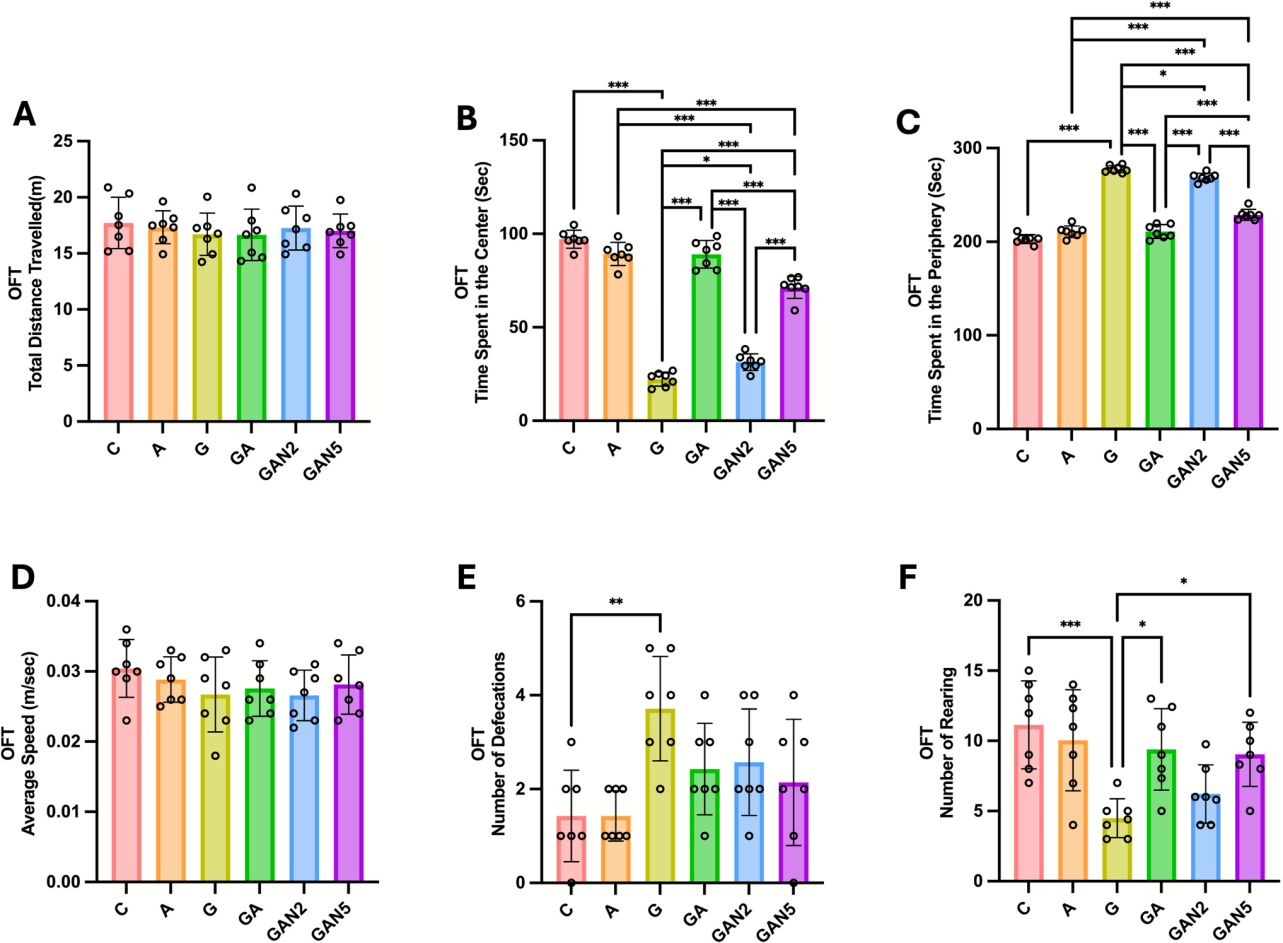
OFT-Total Distance Travelled (m) **(A)**, OFT-Time Spent in the Center (Sec) **(B)**, OFT-Time Spent in the Periphery (Sec) **(C)**, OFT-Average Speed (m/sec) **(D)**, OFT-Number of Defecations **(E)**, OFT-Number of Rearing **(F)** graphs. Statistical analysis: One-way ANOVA followed by Tukey’s multiple comparisons test. Number of animals per group: C (n = 7), A (n = 7), G (n = 7), GA (n = 7), GAN2 (n = 7), GAN5 (n = 7). Data are presented as Mean ± SD. Statistical significance: *p* < 0.05 (*), *p* < 0.01 (**), *p* < 0.001 (***)

One-way ANOVA showed no significant effect of group on OFT-Total Distance Travelled (m) (F(5, 36) = 0.3068, p = 0.91) or OFT-Average Speed (m/sec) (F(5, 36) = 0.8629, p = 0.52). Accordingly, pairwise comparisons revealed no significant differences between the groups (Fig. 2A, 2D). Conversely, a significant group effect was found for OFT-Time Spent in the Center (sec) (F(5, 36) = 236.6, p < 0.001). Pairwise comparisons confirmed significant differences between C and G (mean diff. = 74.90, p < 0.001), A and GAN2 (mean diff. = 57.94, p < 0.001), A and GAN5 (mean diff. = 18.02, p < 0.001), G and GA (mean diff. =  − 66.84, p < 0.001), G and GAN2 (mean diff. =  − 9.06, p = 0.04), G and GAN5 (mean diff. =  − 48.99, p < 0.001), GA and GAN2 (mean diff. = 57.78, p < 0.001), GA and GAN5 (mean diff. = 17.85, p < 0.001), and GAN2 and GAN5 (mean diff. =  − 39.93, p < 0.001) (Fig. 2B). Similarly, OFT-Time Spent in the Periphery (sec) also differed significantly across groups (F(5, 36) = 236.6, p < 0.001). Pairwise comparisons identified significant differences between C and G (mean diff. =  − 74.90, p < 0.001), A and GAN2 (mean diff. =  − 57.94, p < 0.001), A and GAN5 (mean diff. =  − 18.02, p < 0.001), G and GA (mean diff. = 66.84, p < 0.001), G and GAN2 (mean diff. = 9.06, p = 0.04), G and GAN5 (mean diff. = 48.99, p < 0.001), GA and GAN2 (mean diff. =  − 57.78, p < 0.001), GA and GAN5 (mean diff. =  − 17.85, p < 0.001), and GAN2 and GAN5 (mean diff. = 39.93, p < 0.001) (Fig. 2C). For OFT-Number of Defecations, a significant group effect was detected (F(5, 36) = 4.677, p = 0.002), with pairwise comparisons showing a significant difference only between C and G (mean diff. =  − 2.29, p = 0.003) (Fig. 2E). Finally, a significant group effect was observed for OFT-Number of Rearing (F(5, 36) = 6.242, p < 0.001), with significant differences detected between C and G (mean diff. = 6.65, p < 0.001), G and GA (mean diff. =  − 4.91, p = 0.02), and G and GAN5 (mean diff. =  − 4.56, p = 0.03) (Fig. 2F). All p-values were adjusted using Tukey’s post hoc test for multiple comparisons. All data are expressed as mean ± SD and summarized in Table 1.

**Table 1:** Open Field Test results of the experimental groups (mean ± SD)

OFT	C	A	G	GA	GAN2	GAN5
Total Distance Travelled(m)	17,71 ± 2,2	17,33 ± 1,4	16,71 ± 1,8	16,65 ± 2,2	17,26 ± 1,9	17,01 ± 1,4
Time Spent in the Center (Sec)	97,2 ± 4,8	89,3 ± 6,2	22,3 ± 3,7^a***^	89,1 ± 7,3^b***^	31,3 ± 4,4^b*,c,d***^	71,3 ± 5,8^b,c,d,e***^
Time Spent in the Periphery (Sec)	202,8 ± 4,8	210,7 ± 6,2	277,7 ± 3,7^a***^	210,9 ± 7,3^b***^	268,7 ± 4,4^b*,c,d***^	228,7 ± 5,8^b,c,d,e***^
Average Speed (m/sec)	0,03 ± 0,004	0,03 ± 0,003	0,03 ± 0,005	0,03 ± 0,003	0,03 ± 0,003	0,03 ± 0,004
Number of Defecations	1,4 ± 0,9	1,4 ± 0,6	3,7 ± 1,1^a**^	2,4 ± 0,9	2,5 ± 1,1	2,1 ± 1,3
Number of Rearing	11,1 ± 3,1	10,04 ± 3,5	4,5 ± 1,3^a***^	9,4 ± 2,8^b*^	6,22 ± 2,0	9,04 ± 2,2^b*^

C → G, A, a; G → GA, GAN2, GAN5, b; A → GA, GAN2, GAN5, c; GA → GAN2, GAN5, d; GAN2 → GAN5, e. Statistical analysis: One-way ANOVA followed by Tukey’s multiple comparisons test. Number of animals per group: C (n = 7), A (n = 7), G (n = 7), GA (n = 7), GAN2 (n = 7), GAN5 (n = 7). Data are presented as Mean ± SD. Statistical significance: *p* < 0.05 (*), *p* < 0.01 (**), *p* < 0.001 (***).

### NORT Familiarization

**Fig. 3:**
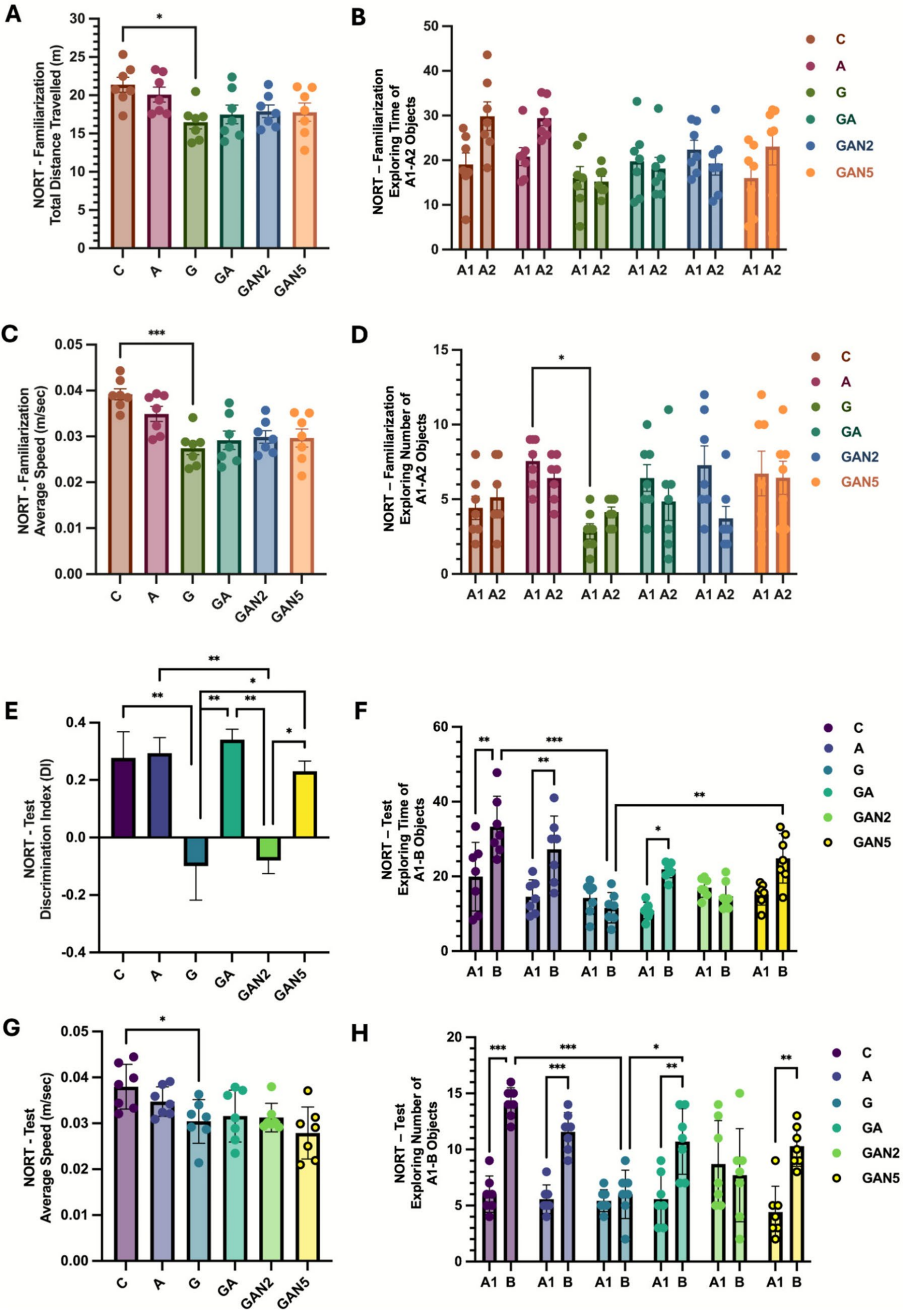
NORT- Familiarization Total Distance Travelled (m) **(A),** NORT- Familiarization Exploring Time of A1-A2 Objects **(B),** NORT- Familiarization Average Speed (m/sec) **(C)**, NORT- Familiarization Exploring Number of A1-A2 Objects **(D),** NORT- Test Discrimination Index (DI) **(E)**, NORT- Test Exploring Time of A1-B Objects **(F)**, NORT- Test Average Speed (m/sec) **(G)**, NORT- Test Exploring Number of A1-B Objects **(H)** graphs. Statistical analysis: Two-way ANOVA with repeated measures followed by Sidak’s multiple comparisons test **(B, D, F, H)** and One-way ANOVA followed by Tukey’s multiple comparisons test **(A, C, E, G).** Statistical analysis: One-way ANOVA followed by Tukey’s multiple comparisons test. Number of animals per group: C (n = 7), A (n = 7), G (n = 7), GA (n = 7), GAN2 (n = 7), GAN5 (n = 7). Data are presented as Mean ± SD. Statistical significance: *p* < 0.05 (*), *p* < 0.01 (**), *p* < 0.001 (***)

One-way ANOVA revealed a significant group effect on NORT-Familiarization Total Distance Travelled (m) (F(5, 36) = 3.240, p = 0.02). Pairwise comparisons showed significant differences only between C and G (mean diff. = 4.896, p = 0.02) (Fig. 3A). Two-way ANOVA showed a significant main effect of group on Exploring Time of A1–A2 objects (column factor: F(5, 72) = 3.690, p = 0.005), a significant main effect of object (row factor: F(1, 72) = 2.559, p = 0.02), and a significant group × object interaction (F(5, 72) = 2.627, p = 0.03) (Fig. 3B), nevertheless the post-hoc pairwise tests showed no significant differences between A1 and A2 within any group. For Average Speed (m/sec), one-way ANOVA indicated a significant group effect (F(5, 36) = 7.372, p < 0.0001). Pairwise comparisons identified significant differences only between C and G (mean diff. = 0.01174, p = 0.0002) (Fig. 3C). Finally, two-way ANOVA for Exploring Number of A1–A2 objects identified a significant main effect of group (column factor: F(5, 72) = 3.598, p = 0.006), while the effect of object (row factor: F(1, 72) = 1.976, p = 0.16) and the group × object interaction (F(5, 72) = 1.735, p = 0.14) were not significant. Pairwise comparisons showed significant differences only between A and G (mean diff. = 4.714, p = 0.03) (Fig. 3D). All data are expressed as mean ± SD and summarized in Table 2.

**Table 2:** Novel Object Recognition Test familiarization phase results (mean ± SD)

NORT-Familiarization	C	A	G	GA	GAN2	GAN5
Total Distance Travelled (m)	21,4 ± 2,6	20,1 ± 2,5	16,5 ± 2,2^a*^	17,5 ± 3,2	17,9 ± 2,1	17,8 ± 3,1
Average Exploring Time of A1-A2 Objects	24.5 ± 7.6	25.1 ± 6.1	15.6 ± 0.6	18.9 ± 1.1	20.9 ± 2.2	19.5 ± 4.9
Average Speed (m/sec)	0,04 ± 0,003	0,03 ± 0,004	0,03 ± 0,003^a***^	0,03 ± 0,005	0,03 ± 0,003	0,03 ± 0,005
Average Exploring Number of A1-A2 Objects	4.7 ± 0.5	7.0 ± 0.8	3.5 ± 0.9	5.6 ± 1.1	5.5 ± 2.5	6.5 ± 0.2

C → G, A, a; G → GA, GAN2, GAN5, b; A → GA, GAN2, GAN5, c; GA → GAN2, GAN5, d; GAN2 → GAN5, e. Statistical analysis: One-way ANOVA followed by Tukey’s multiple comparisons test for Total Distance Travelled (m), Average Speed (m/sec) and Two-way ANOVA with repeated measures followed by Sidak’s multiple comparisons test for Average Exploring Time and Number of A1-A2 Objects. Number of animals per group: C (n = 7), A (n = 7), G (n = 7), GA (n = 7), GAN2 (n = 7), GAN5 (n = 7). Data are presented as Mean ± SD. Statistical significance: *p* < 0.05 (*), *p* < 0.01 (**), *p* < 0.001 (***).

### NORT Test Period

One-way ANOVA indicated a significant group effect on the NORT Test Period Discrimination Index (DI) (F(5, 36) = 7.726, p < 0.001). Pairwise comparisons indicated significant differences between C and G (mean diff. = 0.3759, p = 0.007), A and GAN2 (mean diff. = 0.3734, p = 0.008), G and GA (mean diff. =  − 0.4390, p = 0.001), G and GAN5 (mean diff. =  − 0.3289, p = 0.03), GA and GAN2 (mean diff. = 0.4200, p = 0.002), and GAN2 and GAN5 (mean diff. =  − 0.3098, p = 0.04) (Fig. 3E). Two-way ANOVA showed significant main effects of group (column factor: F(5, 72) = 10.67, p < 0.001) and object (row factor: F(1, 72) = 33.19, p < 0.001) on the Exploring Time of A1–B objects, along with a significant group × object interaction (F(5, 72) = 6.270, p < 0.001). Pairwise comparisons revealed significant differences between A1:C and B:C (mean diff. =  − 13.41, p = 0.002), A1:A and B:A (mean diff. =  − 12.70, p = 0.004), A1:GA and B:GA (mean diff. =  − 11.00, p = 0.03), B:C and B:G (mean diff. = 21.73, p < 0.001), and B:G and B:GAN5 (mean diff. =  − 13.21, p = 0.002) (Fig. 3F). For Average Speed (m/sec), one-way ANOVA indicated a significant group effect (F(5, 36) = 4.024, p = 0.005), with pairwise comparisons showing a significant difference only between C and G (mean diff. = 0.0007, p = 0.048) (Fig. 3G). Finally, two-way ANOVA for the Exploring Number of A1–B objects identified significant main effects of group (column factor: F(5, 72) = 5.012, p < 0.001) and object (row factor: F(1, 72) = 61.79, p < 0.001), as well as a significant group × object interaction (F(5, 72) = 7.598, p < 0.001). Pairwise comparisons indicated significant differences between A1:C and B:C (mean diff. =  − 8.143, p < 0.001), A1:A and B:A (mean diff. =  − 6.000, p < 0.001), A1:GA and B:GA (mean diff. =  − 5.143, p = 0.010), A1:GAN5 and B:GAN5 (mean diff. =  − 5.857, p = 0.001), B:C and B:G (mean diff. = 8.143, p < 0.001), and B:G and B:GA (mean diff. =  − 4.714, p = 0.03) (Fig. 3H). All data are expressed as mean ± SD and summarized in Table 3.

**Table 3:** Novel Object Recognition Test period results (mean ± SD)

NORT-Test	C	A	G	GA	GAN2	GAN5
Discrimination Index (DI)	0,28 ± 0,23	0,29 ± 0,14	−0,09 ± 0,31^a**^	0,34 ± 0,09^b**^	−0,08 ± 0,12^c,d**^	0,23 ± 0,09^b,e*^
Average Exploring Time of A1-B Objects	26.6 ± 9.4	20.9 ± 8.9	12.9 ± 1.8	16.3 ± 7.7	15.8 ± 1.6	20.0 ± 6.7
Average Speed (m/sec)	0,04 ± 0,004	0,03 ± 0,003	0,03 ± 0,004^a*^	0,03 ± 0,005	0,03 ± 0,003	0,03 ± 0,005
Average Exploring Number of A1-B Objects	10.0 ± 5.7	8.5 ± 4.2	5.7 ± 0.4	8.1 ± 3.6	8.2 ± 0.7	7.3 ± 4.1

C → G, A, a; G → GA, GAN2, GAN5, b; A → GA, GAN2, GAN5, c; GA → GAN2, GAN5, d; GAN2 → GAN5, e. Statistical analysis: One-way ANOVA followed by Tukey’s multiple comparisons test for Discrimination Index (DI), Average Speed (m/sec) and Two-way ANOVA with repeated measures followed by Sidak’s multiple comparisons test for Average Exploring Time and Number of A1-B Objects. Number of animals per group: C (n = 7), A (n = 7), G (n = 7), GA (n = 7), GAN2 (n = 7), GAN5 (n = 7). Data are presented as Mean ± SD. Statistical significance: *p* < 0.05 (*), *p* < 0.01 (**), *p* < 0.001 (***).

### MWM-Training

**Fig. 4:**
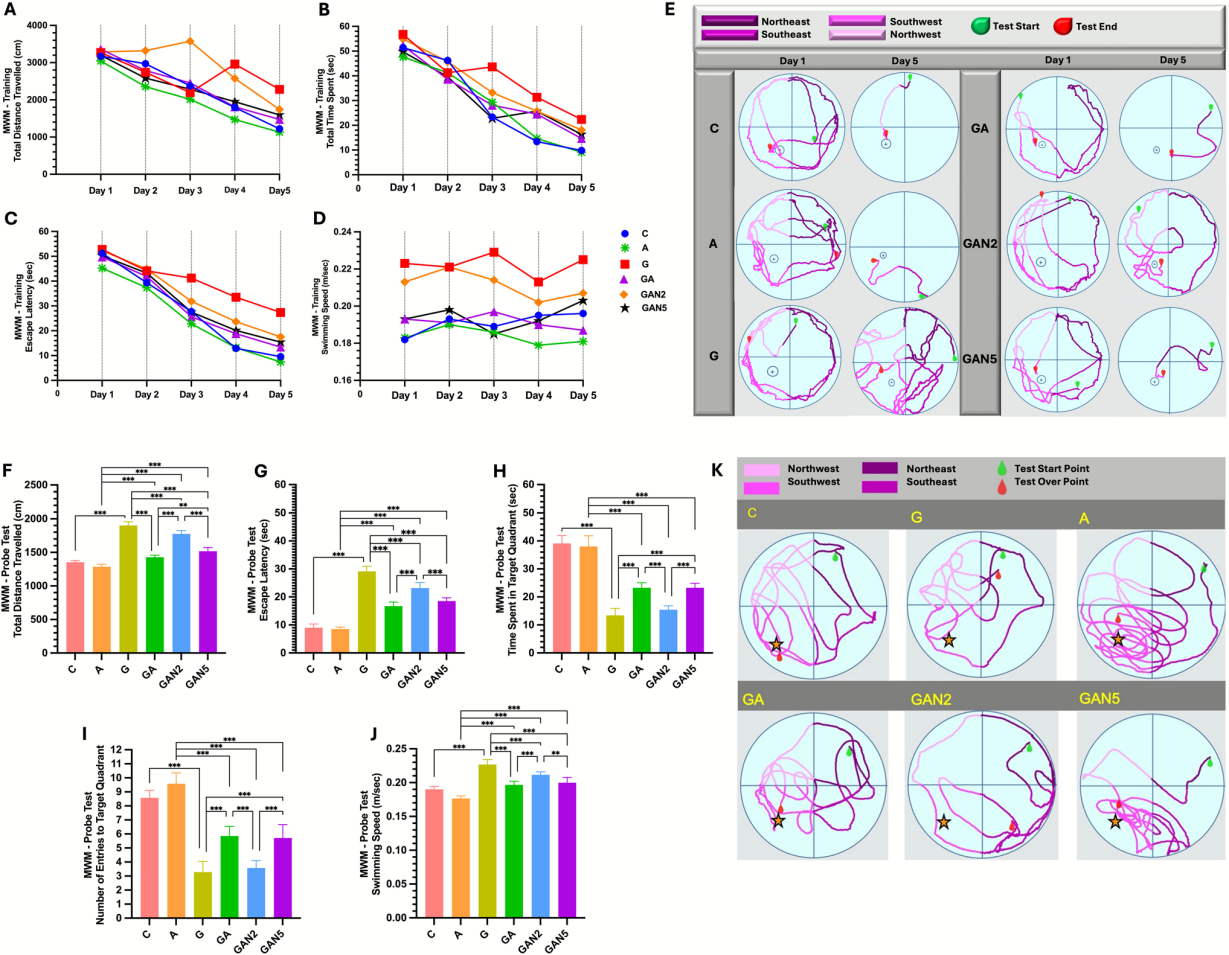
MWM-Training Total Distance Travelled (cm) **(A)**, MWM-Training Total Time Spent (sec) **(B)**, MWM-Training Escape Latency (sec) **(C)**, MWM-Training Swimming Speed (m/sec) **(D)**, MWM-Training Swimming Paths, spatial learning and memory performances of the groups on Day 1 and Day 5 **(E)**, MWM-Probe Test Total Distance Travelled (cm) **(F)**, MWM-Probe Test Escape Latency (sec) **(G)**, MWM-Probe Test Time Spent in Target Quadrant (sec) **(H)**, MWM-Probe Test Number of Entries to Target Quadrant **(I)**, MWM-Probe Test Swimming Speed (m/sec) **(J)**, MWM-Probe Test Swimming Paths **(K)**. Statistical analysis: Two-way Repeated Measures ANOVA followed by Bonferroni’s multiple comparisons test **(A, B, C, D)** and One-way ANOVA followed by Tukey’s multiple comparisons test **(F, G, H, I, J)**. Number of animals per group: C (n = 7), A (n = 7), G (n = 7), GA (n = 7), GAN2 (n = 7), GAN5 (n = 7). Data are presented as Mean ± SD. Statistical significance: *p* < 0.05 (*), *p* < 0.01 (**), *p* < 0.001 (***). **Note:** Swim paths were derived from ANY-maze data and visually enhanced in PowerPoint for clarity; raw tracking data remain unchanged

Two-way repeated measures ANOVA confirmed a significant main effect of group on MWM Training Total Distance Travelled (cm) (column factor: F(5, 20) = 4.820, p = 0.005) and a significant main effect of time (row factor: F(4, 20) = 23.21, p < 0.001). Pairwise comparisons showed a significant decrease (p < 0.01) in all groups on Day 4 compared to Day 1, and a further significant decrease (p < 0.001) on Day 5. Additionally, Total Distance on Day 5 was significantly lower than on Day 2 (p < 0.001) and Day 3 (p < 0.05) (Fig. 4A). Similarly, two-way repeated measures ANOVA found significant main effects of group (column factor: F(5, 20) = 4.995, p = 0.004) and time (row factor: F(4, 20) = 77.03, p < 0.001) on Total Time Spent (sec). Pairwise comparisons indicated a significant decrease (p < 0.001) in all groups on Days 3, 4, and 5 compared to Day 1, and on Days 3, 4, and 5 compared to Day 2. Moreover, a significant decrease (p < 0.001) was observed on Day 5 compared to Day 3 (Fig. 4B). For Escape Latency (sec), two-way repeated measures ANOVA showed significant main effects of group (column factor: F(5, 20) = 13.74, p < 0.001) and time (row factor: F(4, 20) = 136.5, p < 0.001). Pairwise comparisons demonstrated a significant decrease (p < 0.05) on Day 2 compared to Day 1, and a highly significant decrease (p < 0.001) on Days 3, 4, and 5. Additionally, Escape Latency on Days 3, 4, and 5 was significantly lower than on Day 2 (p < 0.001), with further reductions observed on Day 4 compared to Day 3 (p < 0.001) and on Day 5 compared to Day 4 (p < 0.01) (Fig. 4C). Swimming Speed (m/sec) did not differ significantly between groups (column factor: F(5, 20) = 34.37, p < 0.001) nor was there a significant main effect of time (row factor: F(4, 20) = 1.397, p = 0.27), indicating normal locomotor activity throughout the experiment (Fig. 4D). On Day 1, all groups exhibited thigmotaxis and random search strategies. By Day 5, a direct search strategy was observed in group C, an indirect search strategy in groups A and GA, and a directed search strategy in group GAN5. Random search patterns persisted in groups G and GAN2 on Day 5 (Fig. 4E). All data are expressed as mean ± SD and summarized in Table 4.

**Table 4:** 5-day training results of the Morris Water Maze test (mean ± SD)

MWM-Training	C	A	G	GA	GAN2	GAN5
Total Distance Travelled (cm)	2307 ± 810,8	2003 ± 749,8	2690 ± 454,7	2378 ± 754,5	2900 ± 746,9	2323 ± 613,2
Total Time Spent (sec)	28,8 ± 18,9	28,4 ± 16,6	40,1 ± 13,02	31,6 ± 14,3	35,5 ± 14,9	30,8 ± 13,6
Escape Latency (sec)	28,16 ± 17,6	25,2 ± 15,9	39,8 ± 9,8	29,9 ± 15,4	34,1 ± 14,5	31,3 ± 14,9
Swimming Speed (m/sec)	0,19 ± 0,006	0,18 ± 0,004	0,22 ± 0,006	0,19 ± 0,004	0,21 ± 0,007	0,19 ± 0,007

Statistical analysis: Two-way Repeated Measures ANOVA followed by Bonferroni’s multiple comparisons test. Number of animals per group: C (n = 7), A (n = 7), G (n = 7), GA (n = 7), GAN2 (n = 7), GAN5 (n = 7). Data are presented as Mean ± SD.

### MWM-Probe Test

One-way ANOVA highlighted a significant group effect on MWM Probe Test Total Distance Travelled (cm) (F(5, 36) = 209.6, p < 0.001). Pairwise comparisons indicated significant differences between C and G (mean diff. =  − 548.0, p < 0.001), A and GA (mean diff. =  − 140.6, p < 0.001), A and GAN2 (mean diff. =  − 488.4, p < 0.001), A and GAN5 (mean diff. =  − 230.3, p < 0.001), G and GA (mean diff. = 473.3, p < 0.001), G and GAN2 (mean diff. = 125.6, p < 0.001), G and GAN5 (mean diff. = 383.7, p < 0.001), GA and GAN2 (mean diff. =  − 347.8, p < 0.001), GA and GAN5 (mean diff. =  − 89.67, p = 0.007), and GAN2 and GAN5 (mean diff. = 258.1, p < 0.001) (Fig. 4F). One-way ANOVA also indicated a significant group effect on Escape Latency (sec) (F(5, 36) = 211.3, p < 0.001). Pairwise comparisons showed significant differences between C and G (mean diff. =  − 20.06, p < 0.001), A and GA (mean diff. =  − 8.214, p < 0.001), A and GAN2 (mean diff. =  − 14.67, p < 0.001), A and GAN5 (mean diff. =  − 10.01, p < 0.001), G and GA (mean diff. = 12.36, p < 0.001), G and GAN2 (mean diff. = 5.900, p < 0.001), G and GAN5 (mean diff. = 10.56, p < 0.001), GA and GAN2 (mean diff. =  − 6.457, p < 0.001), and GAN2 and GAN5 (mean diff. = 4.657, p < 0.001) (Fig. 4G). Time Spent in Target Quadrant (sec) also differed significantly across groups (F(5, 36) = 137.8, p < 0.001). Pairwise comparisons indicated significant differences between C and G (mean diff. = 25.71, p < 0.001), A and GA (mean diff. = 14.71, p < 0.001), A and GAN2 (mean diff. = 22.57, p < 0.001), A and GAN5 (mean diff. = 14.71, p < 0.001), G and GA (mean diff. =  − 9.857, p < 0.001), G and GAN5 (mean diff. =  − 9.857, p < 0.001), GA and GAN2 (mean diff. = 7.857, p < 0.001), and GAN2 and GAN5 (mean diff. =  − 7.857, p < 0.001) (Fig. 4H). One-way ANOVA showed a significant group effect on the Number of Entries to Target Quadrant (F(5, 36) = 87.35, p < 0.001). Significant pairwise differences were observed between C and G (mean diff. = 5.286, p < 0.001), A and GA (mean diff. = 3.714, p < 0.001), A and GAN2 (mean diff. = 6.000, p < 0.001), A and GAN5 (mean diff. = 3.857, p < 0.001), G and GA (mean diff. =  − 2.571, p < 0.001), G and GAN5 (mean diff. =  − 2.429, p < 0.001), GA and GAN2 (mean diff. = 2.286, p < 0.001), and GAN2 and GAN5 (mean diff. =  − 2.143, p < 0.001) (F4g. 4I). Swimming Speed (m/sec) also showed a significant group effect (F(5, 36) = 65.46, p < 0.001). Pairwise comparisons indicated significant differences between C and G (mean diff. =  − 0.037, p < 0.001), A and GA (mean diff. =  − 0.020, p < 0.001), A and GAN2 (mean diff. =  − 0.035, p < 0.001), A and GAN5 (mean diff. =  − 0.023, p < 0.001), G and GA (mean diff. = 0.030, p < 0.001), G and GAN2 (mean diff. = 0.015, p < 0.001), G and GAN5 (mean diff. = 0.027, p < 0.001), GA and GAN2 (mean diff. =  − 0.015, p < 0.001), and GAN2 and GAN5 (mean diff. = 0.012, p = 0.007) (Fig. 4J). During the 60-s MWM-Probe Test, increased target quadrant entries and a greater search tendency in the target quadrant were observed in the C, A, GA, and GAN5 groups. Conversely, random search patterns persisted in the G and GAN2 groups (Fig. 4K). All data are expressed as mean ± SD and summarized in Table 5.

**Table 5:** Probe trial results of the Morris Water Maze test (mean ± SD)

MWM-Probe	C	A	G	GA	GAN2	GAN5
Total Distance Travelled (cm)	1353 ± 25,5	1287 ± 37,6	1901 ± 56,5^a***^	1428 ± 32^b,c***^	1776 ± 50,3^b,c,d***^	1518 ± 55,8^b,c,e***d**^
Escape Latency (sec)	9,04 ± 1,2	8,5 ± 0,7	29,1 ± 1,8^a***^	16,7 ± 1,4^b,c***^	23,2 ± 1,9^b,c,d***^	18,5 ± 1,1^b,c,e***^
Time Spent in Target Quadrant (sec)	39,1 ± 2,7	38,0 ± 3,8	13,4 ± 2,5^a***^	23,3 ± 1,7^b,c***^	15,4 ± 1,3^c,d***^	23,3 ± 1,6^b,c,e***^
Test Number of Entries to Target Quadrant	8,5 ± 0,5	9,5 ± 0,7	3,2 ± 0,7^a***^	5,8 ± 0,6^b,c***^	3,5 ± 0,5^c,d***^	5,7 ± 0,9^b,c,e***^
Swimming Speed (m/sec)	0,19 ± 0.004	0,17 ± 0,003	0,22 ± 0,01^a***^	0,19 ± 0,01^b,c***^	0,21 ± 0,004^b,c,d***^	0,20 ± 0,01^b,c***,e**^

C → G, A, a; G → GA, GAN2, GAN5, b; A → GA, GAN2, GAN5, c; GA → GAN2, GAN5, d; GAN2 → GAN5, e. Statistical analysis: One-way ANOVA followed by Tukey’s multiple comparisons test. Number of animals per group: C (n = 7), A (n = 7), G (n = 7), GA (n = 7), GAN2 (n = 7), GAN5 (n = 7). Data are presented as Mean ± SD. Statistical significance: *p* < 0.05 (*), *p* < 0.01 (**), *p* < 0.001 (***).

### Biochemical Results

#### Advanced Glycation End Products

**Fig. 5:**
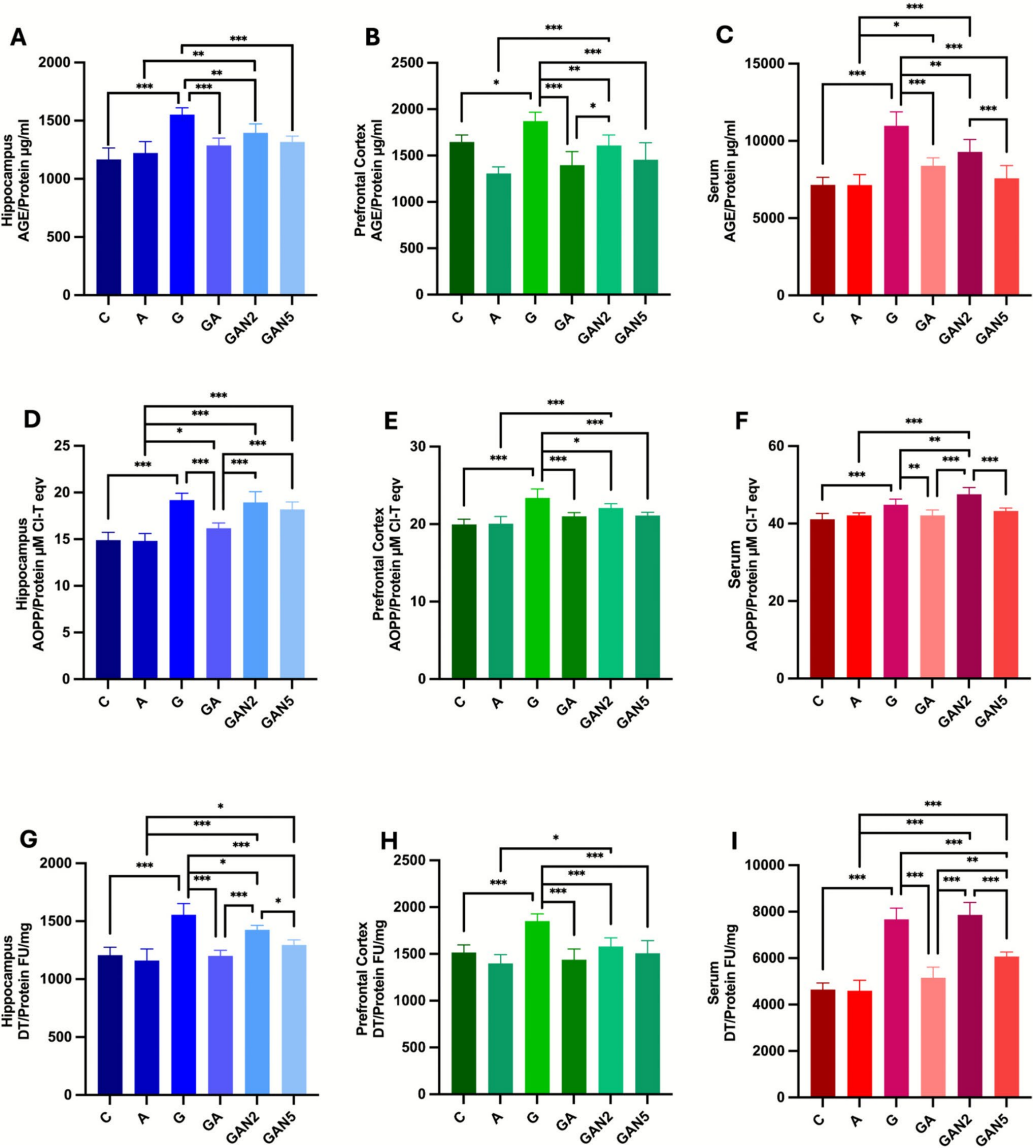
Hippocampus AGE/Protein µg/ml **(A)**, Prefrontal Cortex AGE/Protein µg/ml **(B)**, Serum AGE/Protein µg/ml **(C)**, Hippocampus AOPP/Protein µM Cl-T eqv **(D)**, Prefrontal Cortex AOPP/Protein µM Cl-T eqv **(E)**, Serum AOPP/Protein µM Cl-T eqv **(F)**, Hippocampus DT/Protein FU/mg **(G)**, Prefrontal Cortex DT/Protein FU/mg **(H)**, Serum DT/Protein FU/mg **(I)** graphs. Statistical analysis: One-way ANOVA followed by Tukey’s multiple comparisons test. Number of animals per group: C (n = 7), A (n = 7), G (n = 7), GA (n = 7), GAN2 (n = 7), GAN5 (n = 7). Data are presented as Mean ± SD. Statistical significance: *p* < 0.05 (*), *p* < 0.01 (**), *p* < 0.001 (***)

One-way ANOVA revealed a significant group effect on hippocampal AGE/Protein levels (µg/ml) (F(5, 36) = 22.29, p < 0.001). Pairwise comparisons showed significant differences between C and G (mean diff. =  − 384.9, p < 0.001), A and GAN2 (mean diff. =  − 172.4, p = 0.002), G and GA (mean diff. = 264.4, p < 0.001), G and GAN2 (mean diff. = 156.4, p = 0.006), and G and GAN5 (mean diff. = 234.7, p < 0.001) (Fig. 5A). Similarly, one-way ANOVA indicated a significant group effect on PFC AGE/Protein levels (µg/ml) (F(5, 36) = 19.97, p < 0.001). Significant differences were found between C and G (mean diff. =  − 225.7, p = 0.01), A and GAN2 (mean diff. =  − 301.3, p < 0.001), G and GA (mean diff. = 475.6, p < 0.001), G and GAN2 (mean diff. = 263.0, p = 0.003), G and GAN5 (mean diff. = 415.9, p < 0.001), and GA and GAN2 (mean diff. =  − 212.6, p = 0.02) (Fig. 5B). Finally, one-way ANOVA showed a significant group effect on serum AGE/Protein levels (µg/ml) (F(5, 36) = 30.68, p < 0.001). Pairwise comparisons identificated significant differences between C and G (mean diff. =  − 3824, p < 0.001), A and GA (mean diff. =  − 1243, p = 0.03), A and GAN2 (mean diff. =  − 2151, p < 0.001), G and GA (mean diff. = 2594, p < 0.001), G and GAN2 (mean diff. = 1686, p = 0.001), G and GAN5 (mean diff. = 3410, p < 0.001), and GAN2 and GAN5 (mean diff. = 1724, p < 0.001) (Fig. 5C). All p-values were adjusted using Tukey’s post hoc test for multiple comparisons. All data are expressed as mean ± SD and summarized in Table 6.

**Table 6:** AGE/Protein µg/ml, AOPP/Protein µM Cl-T eqv, DT/Protein FU/mg, KYN/Protein FU/mg, MDA/Protein nmol/mg, T-SH/Protein nmol/mg levels in Hippocampus, Prefrontal Cortex and Serum (mean ± SD)

	**C**	**A**	**G**	**GA**	**GAN2**	**GAN5**
AGE/Protein µg/ml						
Hippocampus	1167 ± 98	1223 ± 98,3	1552 ± 59,3^a***^	1288 ± 64^b***^	1396 ± 77^b**,c**^	1317 ± 50^b***^
PFC	1646 ± 76	1307 ± 70,2	1872 ± 96,4^a*^	1396 ± 146^b***^	1609 ± 113^b**,c***,d*^	1456 ± 183^b***^
Serum	7161 ± 481	7148 ± 668	10,985 ± 895,4^a***^	8391 ± 521,2^b***,c*^	9299 ± 797,1^b**,c***^	7575 ± 836^b,e***^
AOPP/Protein µM Cl-T eqv						
Hippocampus	14,9 ± 0,8	14,8 ± 0,8	19,2 ± 0,7^a***^	17 ± 0,6^b***,c*^	18,6 ± 1,1^c,d***^	18,2 ± 0,8^c,d***^
PFC	19,9 ± 0,7	20,1 ± 0,9	23,4 ± 1,2^a***^	21 ± 0,5^b***^	22,1 ± 0,6^b*,c***^	21,1 ± 0,4^b***^
Serum	41,1 ± 1,5	42,1 ± 0,7	44,9 ± 1,4^a***^	42,1 ± 1,4^b**^	47,6 ± 1,8^b**,c,d***^	43,3 ± 0,7^e***^
DT/Protein FU/mg						
Hippocampus	1208 ± 67,3	1161 ± 99,3	1556 ± 96,8^a***^	1201 ± 48,1^b***^	1426 ± 38,8^b*,c,d***^	1295 ± 43,1^b***,c,e*^
PFC	1515 ± 82	1399 ± 94,3	1850 ± 77,7^a***^	1439 ± 115^b***^	1581 ± 91,8^b***,c*^	1508 ± 136^b***^
Serum	4650 ± 279	4592 ± 452	7674 ± 483^a***^	5154 ± 455,3^b***^	7861 ± 538^c,d***^	6068 ± 193,1^b,c,e***,d**^
KYN/Protein FU/mg						
Hippocampus	569 ± 52	598 ± 55,1	936 ± 23,3^a***^	556 ± 65,4^b***^	867 ± 61^c,d***^	703 ± 28^b,d,e***c**^
PFC	1093 ± 54,4	877 ± 48	1262 ± 87,1^a**^	915,6 ± 60^b***^	1134 ± 98^b*,c,d***^	979,9 ± 67^b***,e**^
Serum	2869 ± 124	2927 ± 112	5270 ± 215^a***^	3423 ± 370^b***,c*^	4851 ± 438^c,d***^	3833 ± 361^b,c,e***^
MDA/Protein nmol/mg						
Hippocampus	5,7 ± 0,51	5,4 ± 0,44	9,8 ± 0,45^a***^	6,5 ± 0,73^b***,c**^	7,9 ± 0,52^b,c,d***^	7,2 ± 0,64^b,c***^
Serum	5,8 ± 0,9	6,8 ± 0,26	15,2 ± 0,85^a***^	7,9 ± 1,02^b***^	13,1 ± 0,97^b**,c,d***^	9,2 ± 0,9^b,c,e***^
T-SH/Protein nmol/mg						
Hippocampus	11,1 ± 0,47	13,4 ± 0,7^a***^	8,6 ± 0,41^a***^	10,4 ± 0,54^b,c***^	9,4 ± 0,5^c***,d*^	10,9 ± 0,7^b,c,e***^
PFC	16,8 ± 0,54	18,4 ± 0,7^a**^	12,9 ± 0,58^a***^	15,8 ± 0,86^b,c***^	14,2 ± 0,8^c***,d**^	15,9 ± 1,1^b,c***,e**^
Serum	22,9 ± 1,3	19,9 ± 2,04	13,73 ± 1,7^a***^	15,9 ± 2,2^c**^	14,8 ± 1,09^c***^	17,6 ± 2,2^b**^

C → G, A, a; G → GA, GAN2, GAN5, b; A → GA, GAN2, GAN5, c; GA → GAN2, GAN5, d; GAN2 → GAN5, e. Statistical analysis: One-way ANOVA followed by Tukey’s multiple comparisons test. Number of animals per group: C (n = 7), A (n = 7), G (n = 7), GA (n = 7), GAN2 (n = 7), GAN5 (n = 7). Data are presented as Mean ± SD. Statistical significance: *p* < 0.05 (*), *p* < 0.01 (**), *p* < 0.001 (***).

#### Advanced Oxidation Protein Products

One-way ANOVA demonstrated a significant group effect on hippocampal AOPP/Protein levels (µM Cl-T eqv) (F(5, 36) = 40.51, p < 0.001). Pairwise comparisons showed significant differences between C and G (mean diff. =  − 4.287, p < 0.001), A and GA (mean diff. =  − 1.352, p = 0.04), A and GAN2 (mean diff. =  − 4.128, p < 0.001), A and GAN5 (mean diff. =  − 3.367, p < 0.001), G and GA (mean diff. = 3.017, p < 0.001), GA and GAN2 (mean diff. =  − 2.776, p < 0.001), and GA and GAN5 (mean diff. =  − 2.015, p < 0.001) (Fig. 5D). Similarly, one-way ANOVA showed a significant group effect on PFC AOPP/Protein levels (µM Cl-T eqv) (F(5, 36) = 21.18, p < 0.001). Significant differences were observed between C and G (mean diff. =  − 3.411, p < 0.001), A and GAN2 (mean diff. =  − 2.022, p < 0.001), G and GA (mean diff. = 2.364, p < 0.001), G and GAN2 (mean diff. = 1.293, p = 0.03), and G and GAN5 (mean diff. = 2.262, p < 0.001) (Fig. 5E). For serum AOPP/Protein levels (µM Cl-T eqv), one-way ANOVA also indicated a significant group effect (F(5, 36) = 22.71, p < 0.001). Pairwise comparisons revealed significant differences between C and G (mean diff. =  − 3.796, p < 0.001), A and GAN2 (mean diff. =  − 5.435, p < 0.001), G and GA (mean diff. = 2.755, p = 0.005), G and GAN2 (mean diff. =  − 2.680, p = 0.006), GA and GAN2 (mean diff. =  − 5.434, p < 0.001), and GAN2 and GAN5 (mean diff. = 4.306, p < 0.001) (Fig. 5F). All p-values were adjusted using Tukey’s post hoc test for multiple comparisons. All data are expressed as mean ± SD and summarized in Table 6.

#### Dityrosine

One-way ANOVA revealed a significant group effect on hippocampal DT/Protein levels (FU/mg) (F(5, 36) = 33.86, p < 0.001). Pairwise comparisons indicated significant differences between C and G (mean diff. =  − 348.7, p < 0.001), A and GAN2 (mean diff. =  − 264.6, p < 0.001), A and GAN5 (mean diff. =  − 134.3, p = 0.01), G and GA (mean diff. = 355.0, p < 0.001), G and GAN2 (mean diff. = 130.7, p = 0.02), G and GAN5 (mean diff. = 261.0, p < 0.001), GA and GAN2 (mean diff. =  − 224.3, p < 0.001), and GAN2 and GAN5 (mean diff. = 130.3, p = 0.02) (Fig. 5G). Similarly, one-way ANOVA identified a significant group effect on PFC DT/Protein levels (FU/mg) (F(5, 36) = 17.57, p < 0.001). Significant differences were observed between C and G (mean diff. =  − 335.0, p < 0.001), A and GAN2 (mean diff. =  − 182.0, p = 0.02), G and GA (mean diff. = 411.1, p < 0.001), G and GAN2 (mean diff. = 269.4, p < 0.001), and G and GAN5 (mean diff. = 342.6, p < 0.001) (Fig. 5H). For serum DT/Protein levels (FU/mg), one-way ANOVA also indicated a significant group effect (F(5, 36) = 86.40, p < 0.001). Pairwise comparisons revealed significant differences between C and G (mean diff. =  − 3024, p < 0.001), A and GAN2 (mean diff. =  − 3269, p < 0.001), A and GAN5 (mean diff. =  − 1476, p < 0.001), G and GA (mean diff. = 2520, p < 0.001), G and GAN5 (mean diff. = 1606, p < 0.001), GA and GAN2 (mean diff. =  − 2708, p < 0.001), GA and GAN5 (mean diff. =  − 914.4, p = 0.003), and GAN2 and GAN5 (mean diff. = 1793, p < 0.001) (F5g. 5I). All p-values were adjusted using Tukey’s post hoc test for multiple comparisons. All data are expressed as mean ± SD and summarized in Table 6.

#### Kynurenine

**Fig. 6:**
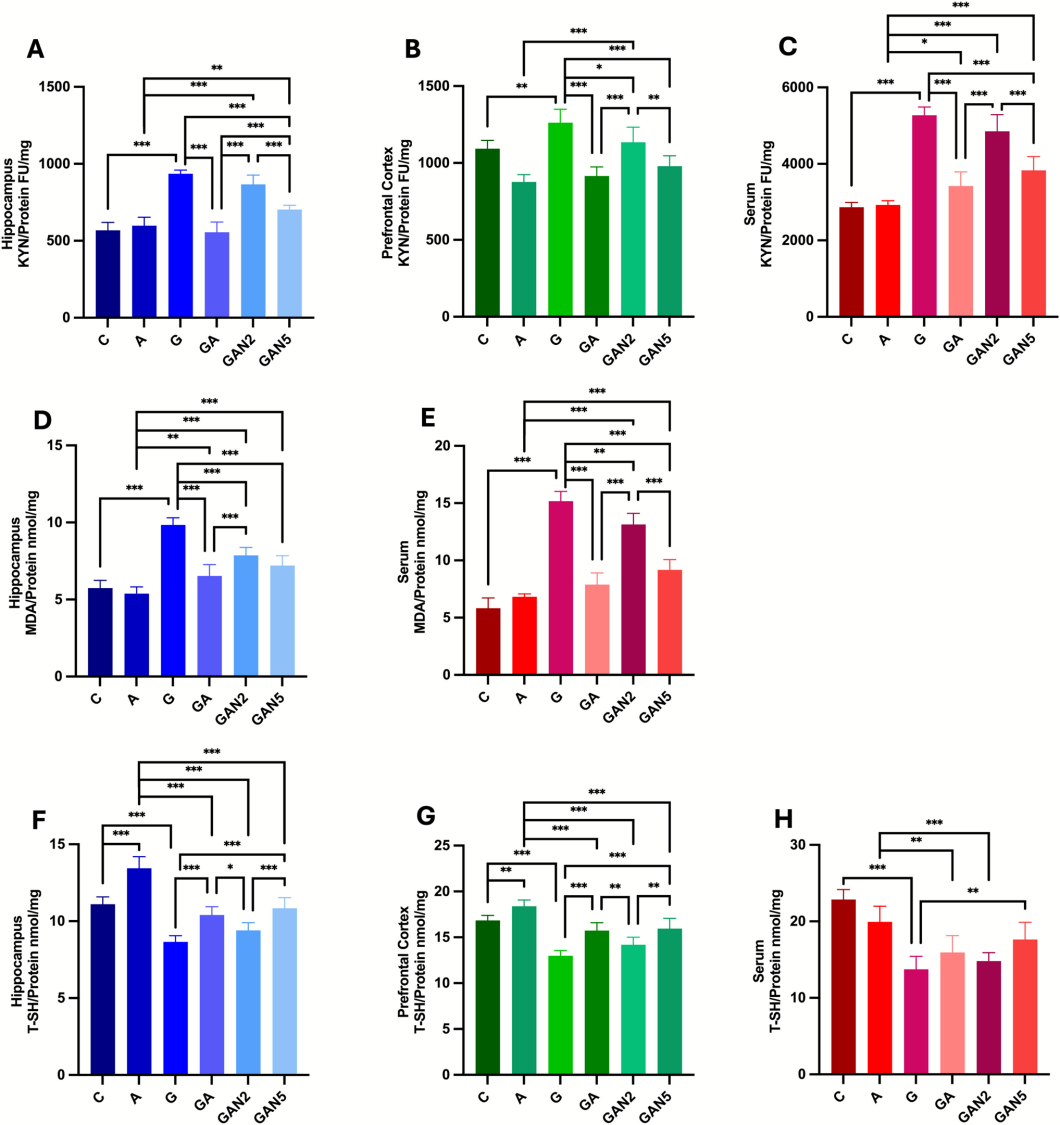
Hippocampus KYN/Protein FU/mg **(A)**, Prefrontal Cortex KYN/Protein FU/mg **(B)**, Serum KYN/Protein FU/mg **(C)**, Hippocampus MDA/Protein nmol/mg **(D)**, Serum MDA/Protein nmol/mg **(E)**, Hippocampus T-SH/Protein nmol/mg **(F)**, Prefrontal Cortex T-SH/Protein nmol/mg **(G)**, Serum T-SH/Protein nmol/mg **(H)** graphs. Statistical analysis: One-way ANOVA followed by Tukey’s multiple comparisons test. Number of animals per group: C (n = 7), A (n = 7), G (n = 7), GA (n = 7), GAN2 (n = 7), GAN5 (n = 7). Data are presented as Mean ± SD. Statistical significance: *p* < 0.05 (*), *p* < 0.01 (**), *p* < 0.001 (***)

One-way ANOVA found a significant group effect on hippocampal KYN/Protein levels (FU/mg) (F(5, 36) = 74.13, p < 0.001). Pairwise comparisons indicated significant differences between C and G (mean diff. =  − 367.1, p < 0.001), A and GAN2 (mean diff. =  − 269.1, p < 0.001), A and GAN5 (mean diff. =  − 105.3, p = 0.004), G and GA (mean diff. = 379.7, p < 0.001), G and GAN5 (mean diff. = 232.9, p < 0.001), GA and GAN2 (mean diff. =  − 310.7, p < 0.001), GA and GAN5 (mean diff. =  − 146.9, p < 0.001), and GAN2 and GAN5 (mean diff. = 163.9, p < 0.001) (Fig. 6A). Similarly, one-way ANOVA identified a significant group effect on PFC KYN/Protein levels (FU/mg) (F(5, 36) = 29.44, p < 0.001). Significant differences were observed between C and G (mean diff. =  − 169.0, p = 0.001), A and GAN2 (mean diff. =  − 256.9, p < 0.001), G and GA (mean diff. = 346.1, p < 0.001), G and GAN2 (mean diff. = 128.1, p = 0.02), G and GAN5 (mean diff. = 281.9, p < 0.001), GA and GAN2 (mean diff. =  − 218.0, p < 0.001), and GAN2 and GAN5 (mean diff. = 153.7, p = 0.003) (Fig. 6B). For serum KYN/Protein levels (FU/mg), one-way ANOVA also indicated a significant group effect (F(5, 36) = 79.04, p < 0.001). Pairwise comparisons showed significant differences between C and G (mean diff. =  − 2401, p < 0.001), A and GA (mean diff. =  − 495.4, p = 0.04), A and GAN2 (mean diff. =  − 1923, p < 0.001), A and GAN5 (mean diff. =  − 905.3, p < 0.001), G and GA (mean diff. = 1848, p < 0.001), G and GAN5 (mean diff. = 1438, p < 0.001), GA and GAN2 (mean diff. =  − 1428, p < 0.001), and GAN2 and GAN5 (mean diff. = 1018, p < 0.001) (Fig. 6C). All p-values were adjusted using Tukey’s post hoc test for multiple comparisons. All data are expressed as mean ± SD and summarized in Table 6.

#### Malondialdehyde

One-way ANOVA revealed a significant group effect on hippocampal MDA/Protein levels (nmol/mg) (F(5, 36) = 59.88, p < 0.001). Pairwise comparisons showed significant differences between C and G (mean diff. =  − 4.106, p < 0.001), A and GA (mean diff. =  − 1.141, p = 0.006), A and GAN2 (mean diff. =  − 2.477, p < 0.001), A and GAN5 (mean diff. =  − 1.823, p < 0.001), G and GA (mean diff. = 3.320, p < 0.001), G and GAN2 (mean diff. = 1.984, p < 0.001), G and GAN5 (mean diff. = 2.638, p < 0.001), and GA and GAN2 (mean diff. =  − 1.336, p < 0.001) (Fig. 6D). Similarly, one-way ANOVA highlighted a significant group effect on serum MDA/Protein levels (nmol/mg) (F(5, 36) = 130.9, p < 0.001). Significant differences were observed between C and G (mean diff. =  − 9.339, p < 0.001), A and GAN2 (mean diff. =  − 6.316, p < 0.001), A and GAN5 (mean diff. =  − 2.361, p < 0.001), G and GA (mean diff. = 7.267, p < 0.001), G and GAN2 (mean diff. = 2.021, p = 0.001), G and GAN5 (mean diff. = 5.976, p < 0.001), GA and GAN2 (mean diff. =  − 5.247, p < 0.001), and GAN2 and GAN5 (mean diff. = 3.955, p < 0.001) (Fig. 6E). All p-values were adjusted using Tukey’s post hoc test for multiple comparisons. All data are expressed as mean ± SD and summarized in Table 6.

#### Total Thiol

One-way ANOVA suggested a significant group effect on hippocampal T-SH/Protein levels (nmol/mg) (F(5, 36) = 59.02, p < 0.001). Pairwise comparisons showed significant differences between C and A (mean diff. =  − 2.337, p < 0.001), C and G (mean diff. = 2.452, p < 0.001), A and GA (mean diff. = 3.036, p < 0.001), A and GAN2 (mean diff. = 4.041, p < 0.001), A and GAN5 (mean diff. = 2.597, p < 0.001), G and GA (mean diff. =  − 1.753, p < 0.001), G and GAN5 (mean diff. =  − 2.193, p < 0.001), GA and GAN2 (mean diff. = 1.005, p = 0.02), and GAN2 and GAN5 (mean diff. =  − 1.445, p < 0.001) (Fig. 6F). Similarly, one-way ANOVA revealed a significant group effect on PFC T-SH/Protein levels (nmol/mg) (F(5, 36) = 41.05, p < 0.001). Significant differences were observed between C and A (mean diff. =  − 1.535, p = 0.010), C and G (mean diff. = 3.862, p < 0.001), A and GA (mean diff. = 2.639, p < 0.001), A and GAN2 (mean diff. = 4.182, p < 0.001), A and GAN5 (mean diff. = 2.426, p < 0.001), G and GA (mean diff. =  − 2.758, p < 0.001), G and GAN5 (mean diff. =  − 2.971, p < 0.001), GA and GAN2 (mean diff. = 1.543, p = 0.010), and GAN2 and GAN5 (mean diff. =  − 1.756, p = 0.002) (Fig. 6G). For serum T-SH/Protein levels (nmol/mg), one-way ANOVA also indicated a significant group effect (F(5, 36) = 24.65, p < 0.001). Pairwise comparisons highlighted significant differences between C and G (mean diff. = 9.125, p < 0.001), A and GA (mean diff. = 3.990, p = 0.003), A and GAN2 (mean diff. = 5.116, p < 0.001), and G and GAN5 (mean diff. =  − 3.918, p = 0.004) (Fig. 6H). All p-values were adjusted using Tukey’s post hoc test for multiple comparisons. All data are expressed as mean ± SD and summarized in Table 6.

### Enzyme-Linked Immunosorbent Assay

#### Active Caspase-3/Protein (ng/mL)

**Fig. 7:**
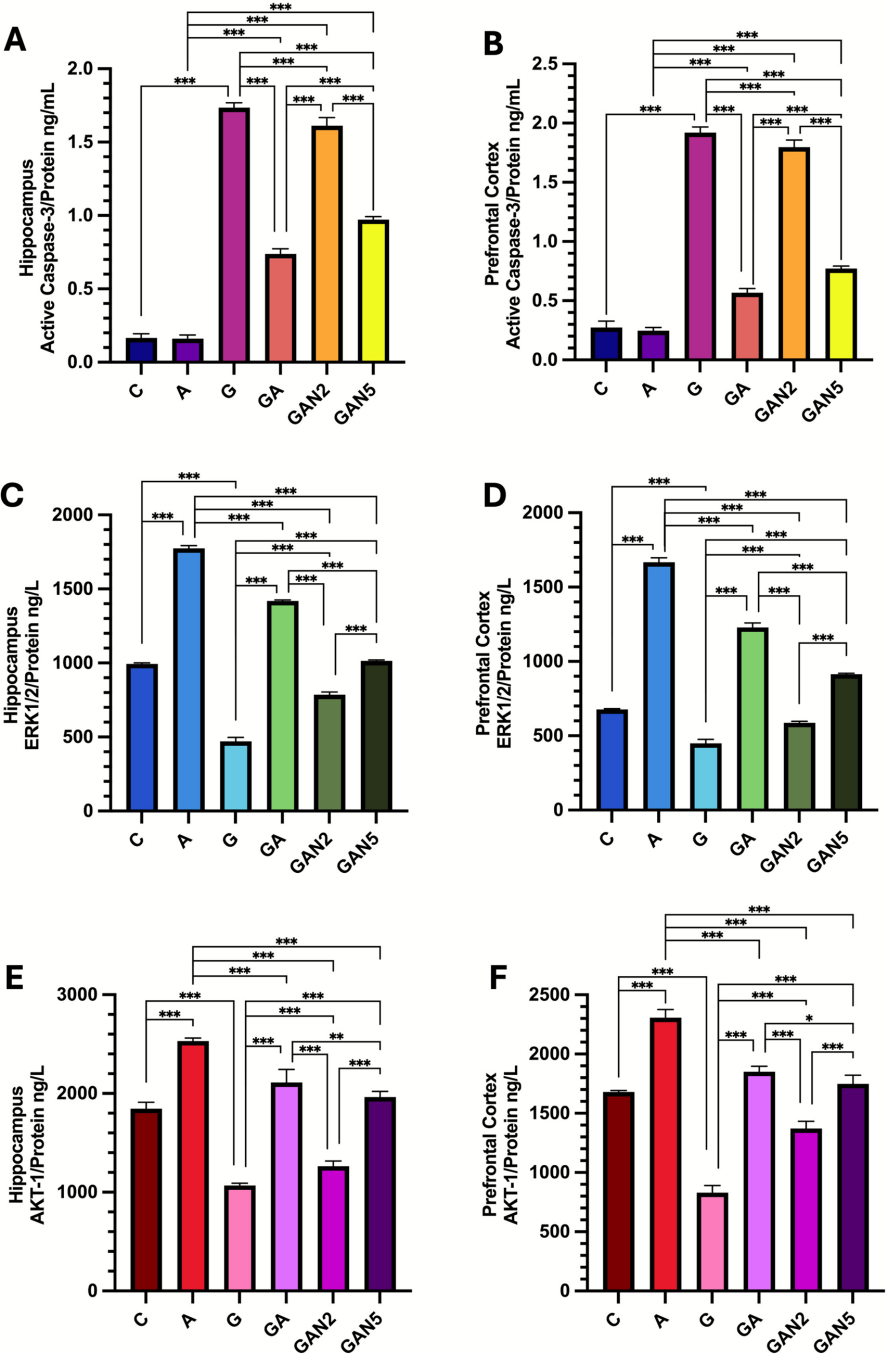
Hippocampus Active Caspase-3/Protein ng/mL **(A)**, Prefrontal Cortex Active Caspase-3/Protein ng/mL **(B)**, Hippocampus ERK1/2/Protein ng/L **(C)**, Prefrontal Cortex ERK1/2/Protein ng/L **(D)**, Hippocampus AKT-1/Protein ng/L **(E)**, Prefrontal Cortex AKT-1/Protein ng/L **(F)** graphs. Statistical analysis: One-way ANOVA followed by Tukey’s multiple comparisons test. Number of animals per group: C (n = 7), A (n = 7), G (n = 7), GA (n = 7), GAN2 (n = 7), GAN5 (n = 7). Data are presented as Mean ± SD. Statistical significance: *p* < 0.05 (*), *p* < 0.01 (**), *p* < 0.001 (***)

One-way ANOVA revealed a significant group effect on hippocampal Active Caspase-3/Protein levels (ng/mL) (F(5, 36) = 2533, p < 0.001). Pairwise comparisons showed significant differences between C and G (mean diff. =  − 1.568, p < 0.001), A and GA (mean diff. =  − 0.5782, p < 0.001), A and GAN2 (mean diff. =  − 1.451, p < 0.001), A and GAN5 (mean diff. =  − 0.8116, p < 0.001), G and GA (mean diff. =  − 1.451, p < 0.001), G and GAN2 (mean diff. =  − 1.753, p < 0.001), G and GAN5 (mean diff. = 0.9962, p < 0.001), GA and GAN2 (mean diff. =  − 0.8730, p < 0.001), GA and GAN5 (mean diff. =  − 0.2334, p < 0.001), and GAN2 and GAN5 (mean diff. = 0.6396, p < 0.001) (Fig. 7A, Table 7). Similarly, one-way ANOVA showed a significant group effect on PFC Active Caspase-3/Protein levels (ng/mL) (F(5, 36) = 2063, p < 0.001). Significant differences were observed between C and G (mean diff. =  − 1.646, p < 0.001), A and GA (mean diff. =  − 0.3196, p < 0.001), A and GAN2 (mean diff. =  − 1.550, p < 0.001), A and GAN5 (mean diff. =  − 0.5245, p < 0.001), G and GA (mean diff. = 1.353, p < 0.001), G and GAN2 (mean diff. = 0.1231, p < 0.001), G and GAN5 (mean diff. = 1.148, p < 0.001), GA and GAN2 (mean diff. =  − 1.230, p < 0.001), GA and GAN5 (mean diff. =  − 0.2049, p < 0.001), and GAN2 and GAN5 (mean diff. = 1.025, p < 0.001) (Fig. 7B, Table 7).

**Table 7:** Active Caspase-3/Protein, ERK1/2 /Protein, AKT-1/Protein levels in Hippocampus and Prefrontal cortex

	**C**	**G**	**A**	**GA**	**GAN2**	**GAN5**
Active Caspase-3/Protein(ng/mL)						
Hippocampus	0,17 ± 0,03	1,73 ± 0,03^a***^	0,16 ± 0.03	0,74 ± 0.04^b,c***^	1,61 ± 0.06^b,c,d***^	0,97 ± 0.02^b,c,d,e***^
PFC	0,27 ± 0,05	1,92 ± 0.04^a***^	0,25 ± 0.03	0,57 ± 0.04^b,c***^	1,80 ± 0.06^b,c,d***^	0,77 ± 0.02^b,c,d,e***^
ERK1/2 /Protein(ng/L)						
Hippocampus	994,4 ± 6,2	471 ± 27.4^a***^	1775 ± 17.9^a***^	1419 ± 6.7^b,c***^	785,1 ± 18.9^b,c,d***^	1014 ± 7.8^b,c,d,e***^
PFC	677,1 ± 4,5	450 ± 26.2^a***^	1667 ± 29.6^a***^	1229 ± 30.6^b,c***^	587,4 ± 10.1^b,c,d***^	914 ± 7.8^b,c,d,e***^
AKT-1/Protein(ng/L)						
Hippocampus	1846 ± 66,4	1068 ± 23^a***^	2531 ± 31^a***^	2112 ± 131.1^b,c***^	1263 ± 53^b,c,d***^	1964 ± 58^b,c,e***,d**^
PFC	1681 ± 12	829,6 ± 60.1^a***^	2307 ± 70^a***^	1850 ± 47^b,c***^	1373 ± 59^b,c,d***^	1749 ± 74^b,c,e***,d*^

C → G, A, a; G → GA, GAN2, GAN5, b; A → GA, GAN2, GAN5, c; GA → GAN2, GAN5, d; GAN2 → GAN5, e. Statistical analysis: One-way ANOVA followed by Tukey’s multiple comparisons test. Number of animals per group: C (n = 7), A (n = 7), G (n = 7), GA (n = 7), GAN2 (n = 7), GAN5 (n = 7). Data are presented as Mean ± SD. Statistical significance: *p* < 0.05 (*), *p* < 0.01 (**), *p* < 0.001 (***).

#### Extracellular Regulated Kinase 1/2/Protein (ng/L)

One-way ANOVA confirmed a significant group effect on hippocampal ERK1/2/Protein levels (ng/L) (F(5, 36) = 5689, p < 0.001). Pairwise comparisons showed significant differences between C and A (mean diff. =  − 780.4, p < 0.001), C and G (mean diff. = 523.9, p < 0.001), A and GA (mean diff. = 355.9, p < 0.001), A and GAN2 (mean diff. = 989.7, p < 0.001), A and GAN5 (mean diff. = 761.3, p < 0.001), G and GA (mean diff. =  − 948.4, p < 0.001), G and GAN2 (mean diff. =  − 314.6, p < 0.001), G and GAN5 (mean diff. =  − 543.0, p < 0.001), GA and GAN2 (mean diff. = 633.9, p < 0.001), GA and GAN5 (mean diff. = 405.4, p < 0.001), and GAN2 and GAN5 (mean diff. =  − 228.4, p < 0.001) (Fig. 7C, Table 7). Similarly, one-way ANOVA showed a significant group effect on PFC ERK1/2/Protein levels (ng/L) (F(5, 36) = 3273, p < 0.001). Significant differences were observed between C and A (mean diff. =  − 990.0, p < 0.001), C and G (mean diff. = 227.7, p < 0.001), A and GA (mean diff. = 438.0, p < 0.001), A and GAN2 (mean diff. = 1080, p < 0.001), A and GAN5 (mean diff. = 753.6, p < 0.001), G and GA (mean diff. =  − 779.7, p < 0.001), G and GAN2 (mean diff. =  − 137.9, p < 0.001), G and GAN5 (mean diff. =  − 464.1, p < 0.001), GA and GAN2 (mean diff. = 641.7, p < 0.001), GA and GAN5 (mean diff. = 315.6, p < 0.001), and GAN2 and GAN5 (mean diff. =  − 326.1, p < 0.001) (Fig. 7D, Table 7).

#### AKT-1/Protein (ng/L)

One-way ANOVA revealed a significant group effect on hippocampal AKT-1/Protein levels (ng/L) (F(5, 36) = 427.3, p < 0.001). Pairwise comparisons showed significant differences between C and A (mean diff. =  − 685.5, p < 0.001), C and G (mean diff. = 777.8, p < 0.001), A and GA (mean diff. = 419.4, p < 0.001), A and GAN2 (mean diff. = 1298, p < 0.001), A and GAN5 (mean diff. = 567.6, p < 0.001), G and GA (mean diff. =  − 1044, p < 0.001), G and GAN2 (mean diff. =  − 195.3, p < 0.001), G and GAN5 (mean diff. =  − 895.6, p < 0.001), GA and GAN2 (mean diff. = 848.6, p < 0.001), GA and GAN5 (mean diff. = 148.2, p = 0.004), and GAN2 and GAN5 (mean diff. =  − 700.4, p < 0.001) (Fig. 7E, Table 7). Similarly, one-way ANOVA revealed a significant group effect on PFC AKT-1/Protein levels (ng/L) (F(5, 36) = 523.2, p < 0.001). Significant differences were observed between C and A (mean diff. =  − 626.2, p < 0.001), C and G (mean diff. = 851.1, p < 0.001), A and GA (mean diff. = 456.6, p < 0.001), A and GAN2 (mean diff. = 934.1, p < 0.001), A and GAN5 (mean diff. = 558.1, p < 0.001), G and GA (mean diff. =  − 1021, p < 0.001), G and GAN2 (mean diff. =  − 543.2, p < 0.001), G and GAN5 (mean diff. =  − 919.3, p < 0.001), GA and GAN2 (mean diff. = 477.5, p < 0.001), GA and GAN5 (mean diff. = 101.4, p = 0.02), and GAN2 and GAN5 (mean diff. =  − 376.1, p < 0.001) (Fig. 7F, Table 7).

### Histological Analyses

**Fig. 8:**
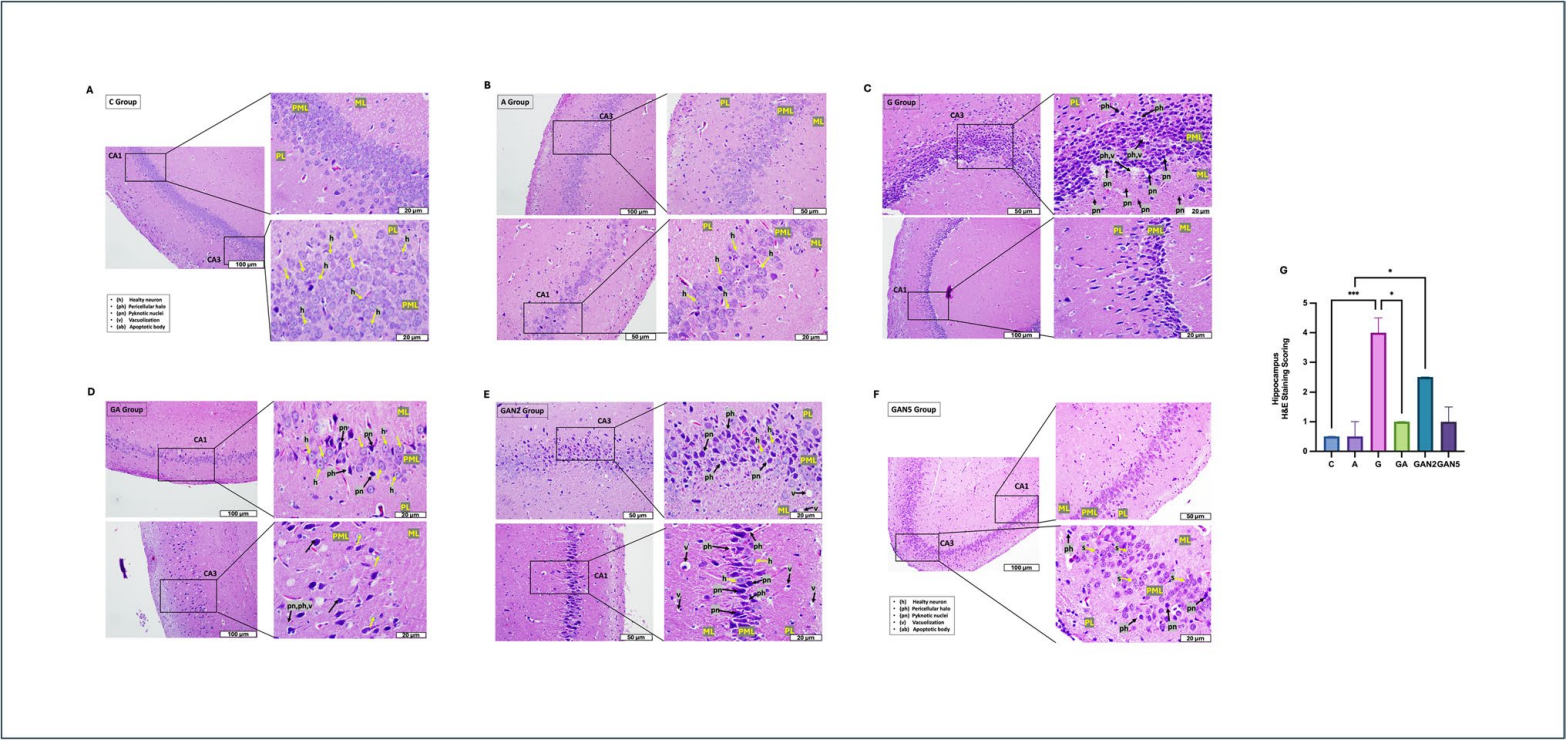
Representative histological images of the hippocampal CA1-CA3 stained with Hematoxylin&Eosin Group C **(A)**, Group A **(B)**, Group G **(C)**, Group GA **(D)**, Group GAN2 **(E)**, Group GAN5 **(F)** are shown. Morphological features include healthy neuron (h), pericellular halo (ph), pyknotic nuclei (pn), vacuolization (v), apoptotic body (ab), molecular layer (ML), pyramidal cell layer (PML), polymorphic layer (PL). Yellow arrows indicate healthy neurons, while black arrows indicate pathological features, including pericellular halos, pyknotic nuclei, vacuolization, and apoptotic bodies. Images are presented at magnifications of 100 µm, 50 µm, and 20 µm to illustrate both general morphology and cellular details for each group. Scale bars are included in all panels. H&E Staining Scoring **(G)** graph is also shown. Statistical analysis: Kruskal–Wallis followed by Dunn’s multiple comparisons test. Number of animals per group: C (n = 7), A (n = 7), G (n = 7), GA (n = 7), GAN2 (n = 7), GAN5 (n = 7). Data are presented as median with interquartile range [IQR]. Statistical significance: *p* < 0.05 (*), *p* < 0.01 (**), *p* < 0.001 (***)

When the hippocampal CA1 and CA3 regions were examined — specifically the molecular layer (ML), polymorphic layer (PML), and pyramidal layer (PL) — healthy (h), light-colored neurons were detected in coronal sections of the C and A groups (Fig. 8A, Fig. 8B). In the G group, neurons exhibiting pericellular halo (ph), indicative of increased edema in PL, as well as dark, shrunken neurons with reduced cytoplasm and pyknotic nuclei (pn), indicative of apoptosis, were evident (Fig. 8C). In the same area, tissue loss consistent with vacuolization (v) was also noted. In the GA group, the occurrence of dark-colored pn and ph findings was reduced (Fig. 8D). In the GAN2 group, ph, pn, and vacuolization were present at a density similar to that observed in the G group (Fig. 8E). In the GAN5 group, healthy neurons appeared at a density comparable to GA, while ph and pn findings were less frequent than in GAN2 (Fig. 8F). Kruskal–Wallis analysis demonstrated a significant group effect on H&E histological scores in the hippocampal CA1 and CA3 regions (H or χ^2^ = 35.51, df = 5, p < 0.001). Pairwise comparisons revealed significant differences between C and G (mean rank diff. =  − 32.64, p < 0.001), A and GAN2 (mean rank diff. =  − 19.36, p = 0.04), and G and GA (mean rank diff. = 21.07, p = 0.02) (Fig. 8G). All data are expressed as median [(25%percentile-75% Percentile) IQR] and summarized in Table 8.

**Table 8:** Hippocampal Hematoxylin&Eosin and Prefrontal Cortex Cresyl Violet staining results (median [IQR])

	**C**	**A**	**G**	**GA**	**GAN2**	**GAN5**
Hippocampal H&E staining	0.5 [0–0.5]	0.5 [0.5–1.0]	4.0 [3.5–4.5]^a***^	1.0 [0.5–1.0]^b*^	2,5 [2,0–2,5]^c*^	1,0 [1,0–1.5]
PFC cresyl violet staining	0.5 [0–0.5]	0.5 [0,5–1,0]	3,5 [3.0–4.0]^a***^	1.0 [1.0–1.5]	3.0 [3.0–3.5]^c**^	2.0 [1.5–2.0]

C → G, A, a; G → GA, GAN2, GAN5, b; A → GA, GAN2, GAN5, c; GA → GAN2, GAN5, d; GAN2 → GAN5, e. Statistical analysis: Kruskal–Wallis followed by Dunn’s multiple comparisons test. Number of animals per group: C (n = 7), A (n = 7), G (n = 7), GA (n = 7), GAN2 (n = 7), GAN5 (n = 7). Data are presented as median [(25%percentile-75% Percentile) IQR].

**Fig. 9:**
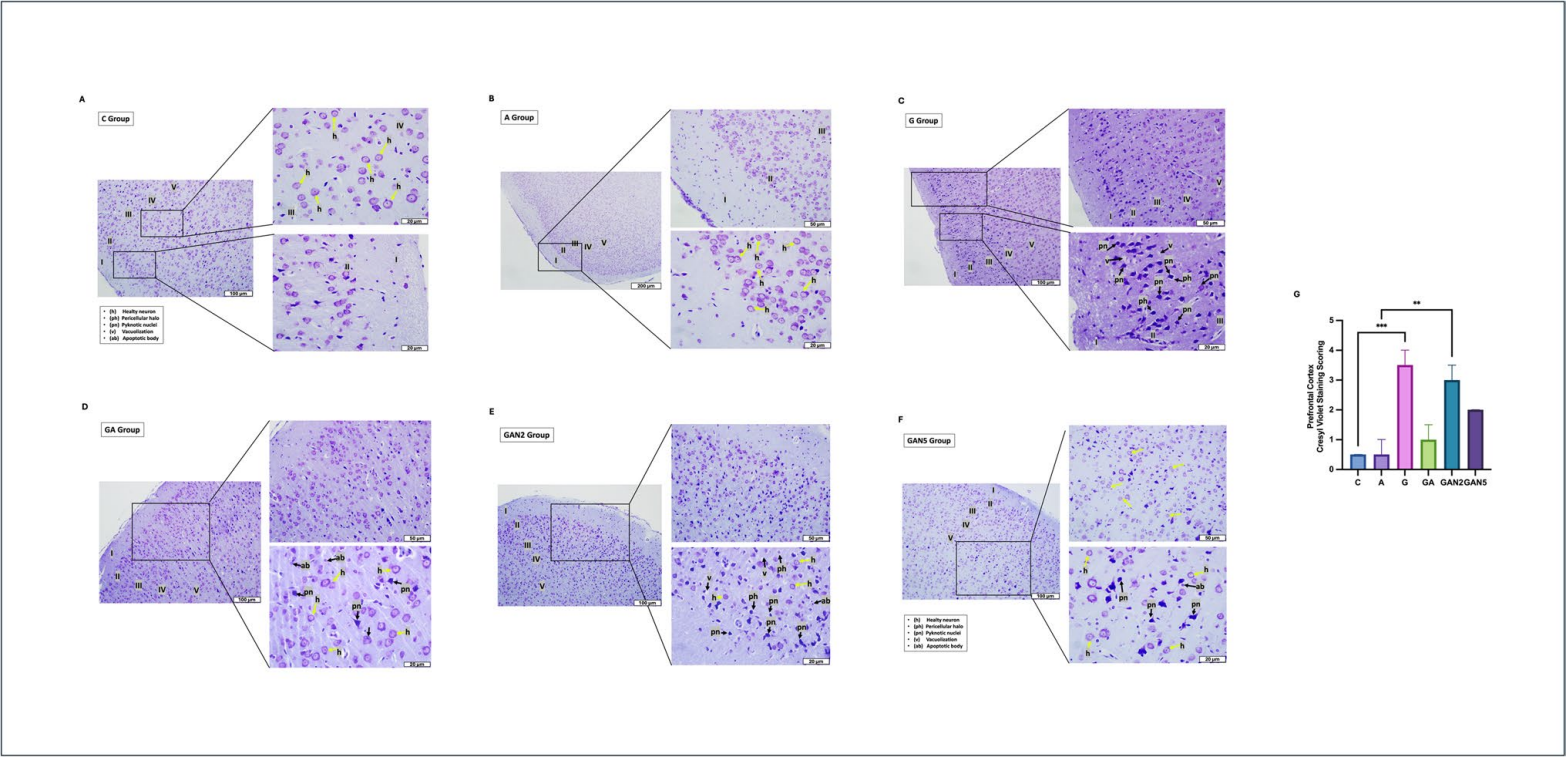
Representative histological images of the Prefrontal Cortex stained with Cresyl Violet Group C **(A)**, Group A **(B)**, Group G **(C)**, Group GA **(D)**, Group GAN2 **(E)**, Group GAN5 **(F)** are shown. Morphological features includes healty neuron (h), pericellular halo (ph), pyknotic nuclei (pn), vacuolization (v), apoptotic bodies (ab), Molecular (I), external granular (II), external pyramidal (III), internal granular (IV), internal pyramidal (V) and polymorphic layer (VI). Yellow arrows indicate healthy neurons, while black arrows indicate pathological features, including pericellular halos, pyknotic nuclei, vacuolization, and apoptotic bodies. Images are presented at magnifications of 100 µm, 50 µm, and 20 µm to illustrate both general morphology and cellular details for each group. Scale bars are included in all panels. Cresyl Violet Staining Scoring **(G)** graph is also shown. Statistical analysis: Kruskal–Wallis followed by Dunn’s multiple comparisons test. Number of animals per group: C (n = 7), A (n = 7), G (n = 7), GA (n = 7), GAN2 (n = 7), GAN5 (n = 7). Data are presented as median with interquartile range [IQR]. Statistical significance: *p* < 0.05 (*), *p* < 0.01 (**), *p* < 0.001 (***)

Among the layers of the prefrontal cortex (PFC) — molecular (I), external granular (II), external pyramidal (III), internal granular (IV), internal pyramidal (V), and polymorphic (VI) — layers II, III, IV, and V were evaluated. In parasagittal sections of the C and A groups, homogeneously distributed normocytic neurons with intact vesicular nuclei were identified (Fig. 9A, Fig. 9B). In the G group, neurons exhibiting pyknotic nuclei (pn), pericellular halo (ph), and vacuolization (v) were evident (Fig. 9C). In the GA group, diffusely distributed small amounts of pn and ph along with moderately healthy neurons were noted (Fig. 9D). In GAN2, diffuse presence of pn, ph, v, apoptotic bodies (ab), and sparse healthy neurons were detected in layers II, III, and IV (Fig. 9E). In GAN5, small amounts of pn, ph, and ab were observed, along with a greater number of healthy neurons (Fig. 9F). Kruskal–Wallis analysis demonstrated a significant group effect on Cresyl Violet histological scores in the PFC (H or χ^2^ = 37.13, df = 5, p < 0.001). Pairwise comparisons showed significant differences between C and G (mean rank diff. =  − 30.86, p < 0.001) and between A and GAN2 (mean rank diff. =  − 23.86, p = 0.003)(Fig. 9G). All data are expressed as median [(25%percentile-75% Percentile) IQR] and summarized in Table 8.

## Dıscussıon

In this study, we established an excitotoxicity model in male rats using D-glutamic acid and investigated the neuroprotective and cognitive effects of exogenous Apelin-13, focusing on the potential involvement of NPY2R and NPY5R pathways.

Caspase-3, a key effector in the apoptotic pathway, is activated during excitotoxicity and leads to DNA fragmentation and neuronal death [96–98]. Previous studies have shown that Apelin-13 treatment reduces active caspase-3 levels and suppresses apoptosis in excitotoxicity and subarachnoid hemorrhage models [99, 100]. Similarly, in Alzheimer’s disease (AD), excitotoxicity, and ischemic stroke models, NPY2R activation—along with a lesser contribution from NPY5R—attenuated caspase-3 activation, suppressed glutamate release, increased AKT phosphorylation, and preserved neuronal integrity [33, 101, 102]. Consistent with these findings, our results show that blocking NPY2R significantly diminishes the neuroprotective effects of Apelin-13, highlighting the predominant role of NPY2R, with NPY5R having a more limited effect.

In AD, PI3K/AKT signaling is disrupted by increased caspase-3 activation [103]. In our study, excitotoxicity in the G group increased active caspase-3, reduced phosphorylated ERK1/2 and AKT-1 levels, and induced mitochondrial dysfunction and oxidative stress, whereas Apelin-13 treatment in the GA group reversed these changes.

ERK1/2, a pro-survival kinase in the Ras-Raf-MEK-ERK cascade, regulates key processes such as adhesion, migration, and transcription [104]. Excitotoxic overstimulation of NMDARs has been shown to inhibit ERK1/2 phosphorylation, thereby reducing neuronal survival [105–108]. In our model, D-glutamic acid suppressed ERK1/2 activation, while Apelin-13 restored it, likely by inhibiting NMDARs and activating endogenous apelinergic signaling [109]. The increased ERK1/2 phosphorylation observed in the A group relative to C suggests that exogenous Apelin-13 enhances this endogenous signaling.

In addition to ERK1/2, excitotoxicity also impaired AKT signaling and promoted apoptosis. Previous studies have demonstrated that NPY2R activation suppresses apoptosis by increasing ERK1/2 and AKT phosphorylation [25]. In line with this, we observed that Apelin-13 inhibited active caspase-3 activation and promoted ERK1/2 and AKT-1 signaling, conferring neuroprotection predominantly through NPY2R and, to a lesser extent, NPY5R. When both receptors were blocked, Apelin-13 lost its protective effect.

AKT, a serine/threonine kinase, regulates key processes such as proliferation, differentiation, and cell survival. Its dysfunction has been implicated in neurodegenerative, cardiovascular, inflammatory, autoimmune diseases, and cancer [110]. In excitotoxicity, reduced phosphorylated AKT and increased neuronal death have been reported in the hippocampal CA1–CA3 regions [111]. Alongside suppression of ERK1/2 and AKT phosphorylation, excitotoxicity also triggers excessive ROS production and cytochrome c release. Apelin-13 has been shown to counteract apoptosis and excitotoxic death via APJ/Gi-Gq signaling [112]. In our study, Apelin-13 enhanced AKT-1 activation, which helped preserve mitochondrial integrity, inhibited apoptosis, and protected against excitotoxic damage.

NPY contributes to neuroprotection by inhibiting glutamatergic transmission in pyramidal neurons. This occurs through VGCC blockade mediated by NPY2Rs, which are highly expressed at the presynaptic terminals of mossy fibers in CA3 and Schaffer collaterals in CA1 [113–117]. In PD models, NPY2R agonists have been shown to protect against excitotoxicity by activating both ERK1/2 and PI3K/AKT pathways in cortical glutamatergic afferents [38]. Similarly, higher NPY2R gene expression has been associated with delayed onset of Huntington’s disease (HD), suggesting a potential disease-modifying effect [118]. In HD models, NPY agonists improved neuronal survival by enhancing ERK1/2 phosphorylation downstream of NPY2R activation [119]. In a rat excitotoxicity model, NPY2R agonists protected pyramidal neurons in DG, CA1, and CA3, whereas NPY5R agonists conferred protection only in DG and CA3, without effect in CA1 [120]. This protective effect was abolished by selective NPY2R antagonists (e.g., BIIE0246), underscoring the central role of NPY2R [121–123]. In neurodegenerative conditions characterized by elevated presynaptic Ca^2^⁺, NPY2R activation suppresses glutamate release via VGCC inhibition and promotes neuronal survival by modulating ERK1/2 and PI3K/AKT pathways. Apelin-13 provides neuroprotection against excitotoxicity by suppressing NMDAR/AMPAR activity through the activation of the ERK1/2 and PI3K/AKT pathways [22, 23]. Our findings further support the dominant contribution of NPY2R to Apelin‑13’s neuroprotective effects compared to NPY5R, likely due to its autoregulatory role through presynaptic negative feedback. The inability of GAN5 to reach GA levels despite intact NPY2R activity, together with the modest improvement seen in GAN2 compared to G, supports the interpretation that NPY5R provides a small yet measurable auxiliary contribution. In the methodology, instead of the lowest effective dose in the literature (3 mg/kg) [77] we used 1.5 mg/kg of the NPY5R antagonist to ensure dose consistency and comparability with the NPY2R antagonist. However, in the GAN2 group, where no intervention on NPY5R was made, the reduced protective effect clearly demonstrates that our use of the 1.5 mg/kg NPY5R antagonist dose is not responsible for the reduced protection.

OFT provides insight into locomotor activity and the general anxiety-related behavior of animals [124]. Previous studies showed that Apelin-13 treatment in a chronic social defeat stress model did not alter total movement distance in OFT or produce stress–drug interactions [125]. Conversely, in depression models, decreased NPY2R expression in the PFC was associated with depressive-like behaviors [126]. In our study, the average total distance (s) and average speed (m/s) in all groups were comparable to the control group, supporting the absence of locomotor deficits. However, increased thigmotaxis and defecation, along with suppressed exploratory behavior, reduced motivation, and diminished responsiveness to environmental stimuli in the G and GAN2 groups reflected emotional disturbances driven by glutamate-induced excitotoxicity. In contrast, the lack of anxiety-related behaviors in the C and A groups suggests that Apelin-13 does not alter behavior in healthy animals but can ameliorate injury-induced behavioral disturbances. Notably, NPY2R antagonism exacerbated the behavioral impairments, highlighting the pivotal role of NPY2R in mediating Apelin-13’s therapeutic effects. Notably, a recent study using the Y2R agonist NPY13‑36 in a rat model of cerebral ischemia reported similar findings, where motor function and infarct size improved, yet spontaneous mobility in the open field test did not significantly change [127]. In contrast, our findings demonstrate that Apelin‑13 ameliorated OFT deficits through a mechanism requiring intact NPY2R signaling, suggesting that while Y2R activation alone may not suffice to enhance spontaneous exploratory behavior, it is nevertheless a critical component of the Apelin‑13–mediated neuroprotective pathway restoring behavioral performance after excitotoxic injury.

NORT is commonly used to assess recognition memory and short-term memory (STM) [128], relying on both prefrontal cortex (PFC) and hippocampal integrity [129]. Performance in NORT is sensitive to hippocampal and cortical lesions [130], and excitotoxic injury disrupts neuronal structure and function in these regions [12]. Previous studies have shown that Apelin-13 treatment improves memory deficits observed in NORT under depressive conditions, while it does not alter recognition memory in healthy animals. Moreover, blocking PI3K/AKT and ERK1/2 pathways abolished Apelin-13’s protective effects [23]. Similarly, in stressed animals, decreased discrimination index (DI) was reversed by Apelin-13 treatment without affecting total exploration time [129]. In a PD model, excitotoxic damage impaired recognition of novel objects, whereas Apelin-13 increased DI, suggesting improved cognitive performance [131]. Activation of exogenous NPY receptors has also been shown to enhance recognition memory recall [132]. Although no differences in average speed were observed in the open-field test, a statistically significant but mild reduction was detected only in the G group during the NORT (p < 0.05). No significant differences were found among the other groups, and the absence of changes in the open-field test suggests that this reduction is unlikely to reflect a general locomotor impairment. Instead, it may be attributed to glutamate-induced excitotoxic damage, resulting in reduced exploratory drive, impaired cognitive engagement, or individual variability due to transient stress, heightened environmental reactivity, or low spontaneous activity observed in some animals during testing. Moreover, as suggested by previous studies, the novel object or environment may induce a mild neophobic response in some animals, leading to cautious behavior and reduced locomotion during the test [133].

The C, A, and GA groups demonstrated healthy STM by preferentially exploring the novel object during testing. In contrast, the G, GAN2 groups spent equal time on both objects, indicating impaired STM, consolidation, and memory discrimination. The negative DI observed in the G and GAN2 groups reflects pronounced memory damage under excitotoxic conditions, whereas the positive DI in the GAN5 group, similar to GA, supports the neuroprotective role of Apelin-13 on cognitive function, mediated predominantly via NPY2R activation.

MWM is a well-established test for evaluating spatial memory, learning, and long-term memory. Spatial reference memory allows animals to locate a hidden platform by integrating egocentric and allocentric navigation strategies through repeated exploration of stable environmental cues [134, 135]. This ability depends on the hippocampal–PFC circuitry, which facilitates route planning and efficient target-oriented navigation [130]. Disruption of this circuitry impairs spatial mapping and learning performance [136–138]. In our study, total distance traveled, a key indicator of spatial learning and memory [81, 139], demonstrated that animals in the G and GAN2 groups displayed impaired performance, favoring complex and inefficient routes during training. These findings highlight the detrimental effects of glutamate-induced excitotoxicity on spatial memory. Moreover, the beneficial effects of Apelin-13 on learning and memory were significantly attenuated when NPY2R was blocked, as reflected by the poorer performance of the GAN2 group compared to the GAN5 group. The superior performance of GAN5 relative to GAN2 suggests a predominant role of NPY2R over NPY5R in mediating Apelin-13’s neuroprotective effects. The preference for short and direct routes in the A and GA groups further underscores Apelin-13’s capacity to preserve spatial reference memory in the setting of excitotoxic injury.

Hippocampal damage in striatal regions has been shown to promote compensatory egocentric navigation strategies [140, 141]. Consistently, animals in the G and GAN2 groups predominantly used non-spatial, egocentric patterns such as thigmotaxis, scanning, and random search, which are independent of hippocampal-based allocentric learning, indicating impaired allocentric memory mapping. In excitotoxic conditions, the conversion of short-term memory (STM) to long-term memory (LTM) was disrupted, and allocentric mapping was compromised, leading to reliance on adaptive egocentric strategies. In contrast, the C, A, GA, and GAN5 groups displayed goal-directed, direct, focal, and indirect search strategies, consistent with preserved hippocampal learning, intact allocentric spatial mapping, and maintained LTM, confirming the protective effects of Apelin-13 through NPY2R and NPY5R pathways.

Although studies investigating the role of NPY5R in spatial memory are limited, it has been reported that mice with NPY1 receptor knockout in hippocampal CA1 and CA3 neurons expressing NPY5R, largely used focal and directed allocenctric search strategies in the MWM probe test, indicating a role in enhancing spatial memory. [18, 142].

Previous studies reported that damage to hippocampal integrity and spatial memory function adversely affects total distance traveled and escape latency in both MWM training and probe trials [143]. Our histological analyses further demonstrated that extensive neuronal damage in the CA1, CA3, and PFC regions in the G and GAN2 groups impaired cell-based spatial mapping, whereas preserved cellular and tissue integrity in the GAN5 and GA groups reinforced the therapeutic importance of Apelin-13, mediated primarily by NPY2R and, to a lesser extent, by NPY5R as supported by both the significance levels and the effect sizes. In line with neurotoxicity models, our findings also revealed increased escape latency and total distance, and decreased time spent and entries into the target quadrant during MWM training and probe trials in the G and GAN2 groups [99, 131]. Similar deficits were observed in total time, swimming speed, and escape latency during training. During the probe trials, the G and GAN2 groups exhibited reduced time and entries in the target quadrant and increased total distance and escape latency due to excitotoxic damage, whereas Apelin-13 ameliorated these impairments.

Oxidative stress resulting from excessive ROS production is a key contributor to neurodegenerative diseases through cumulative oxidative damage. ROS overproduction promotes DNA methylation and neurotoxicity by increasing levels of oxidative markers such as AGE, AOPP, KYN, DT, and MDA, while depleting antioxidant molecules such as T‑SH [144, 145]. AGE, AOPP, and MDA are well‑established markers of oxidative damage, associated with mitochondrial dysfunction, glutamate dysregulation, and apoptotic signaling via pathways such as RAGE–ERK1/2 activation [146–154]. In our study, the excitotoxic G group exhibited significantly elevated AGE, AOPP, and MDA levels alongside suppressed ERK1/2 and AKT activity and increased caspase‑3 activation, indicative of oxidative damage and apoptosis. Apelin‑13 treatment in the A and GA groups reduced these markers and restored signaling, consistent with its antioxidative potential [155]. Similarly, DT and KYN, which contribute to oxidative injury through protein cross‑linking and glutamate‑mediated excitotoxicity respectively, were also elevated in the G group and mitigated by Apelin‑13 treatment [156–160]. The decline in these markers suggests a broader suppression of ROS‑driven damage. Finally, the marked reduction in T‑SH levels in the G and GAN2 groups reflects excessive oxidation of sulfhydryl groups, impairing antioxidant defenses [161–164]. Restoration of T‑SH in the GA group further supports the antioxidant and neuroprotective role of Apelin‑13 via NPY2R and, to a lesser extent, NPY5R signaling [155]. These biochemical improvements paralleled better cognitive and behavioral outcomes, reinforcing that attenuation of oxidative stress is one of the downstream mechanisms mediating the neuroprotective effects of Apelin‑13 through NPY2R and NPY5R pathways.

Consistent with these findings, our data showed that increased T-SH levels in the GA group indicate activation of the antioxidant defense system, protection against oxidative stress and mitochondrial dysfunction, and reduced ROS generation. The synergistic action of Apelin-13 with NPY2R and NPY5R signaling pathways was also corroborated by biochemical markers. Importantly, behavioral outcomes in MWM, NORT, and OFT tests were aligned with changes in oxidative stress parameters, reinforcing the link between oxidative damage mitigation and cognitive improvement.

Histopathologically, neurodegeneration is marked by the presence of darkly stained pyknotic nuclei (pn) indicative of apoptosis, loss of membrane integrity, pericellular halo (ph) reflecting severe neuronal edema, and intracellular foamy vacuolization signaling oxidative stress and toxicity [165, 166]. In the G and GAN2 groups of our study, these pathological features were evident despite preserved membrane integrity, accompanied by increased caspase-3, confirming ongoing apoptosis [167]. In PD models, early reductions in CA1 LTP and associated neuronal damage have been ameliorated by Apelin-13 administration, supporting its neuroprotective role [168].

This may reflect region‑specific and pathway‑selective roles of NPY5R, particularly in the dentate gyrus and CA3 regions, as reported in previous studies. The partial enhancement seen with NPY5R antagonism could be attributed to its involvement in modulating neurogenesis, synaptic plasticity, and excitatory–inhibitory balance through distinct downstream mechanisms. These findings also may reflect receptor–receptor crosstalk between NPY2R and NPY5R and their potential interaction with the apelinergic system through heterodimerization or shared downstream pathways such as PI3K/AKT and ERK1/2 signaling. Heterodimerization of G‑protein–coupled receptors (GPCRs) has been reported in other neuropeptide systems, suggesting that such complexes may modulate ligand affinity, signaling bias, or regional specificity of the neuroprotective response. Future studies should investigate whether NPY receptors and APJ colocalize and form heteromeric complexes, which could help elucidate these mechanisms.

A limitation of this study is that receptor function was inferred solely from pharmacological blockade using selective antagonists, without direct assessment of NPY2R and NPY5R expression levels, localization, or downstream receptor‑specific signaling changes. Due to insufficient tissue volume, MDA levels could not be measured in the prefrontal cortex. Techniques such as polymerase-chain reaction (PCR), Western blotting, immunohistochemistry, or phospho‑specific antibodies could provide additional insights. Future studies should aim to directly characterize these receptors’ distribution and signaling dynamics to strengthen the mechanistic understanding of their roles in Apelin‑13–mediated neuroprotection.

Despite these limitations, our findings highlight the potential of Apelin‑13 as a neuroprotective agent targeting excitotoxic and oxidative pathways, with NPY2R and NPY5R contributing distinctly to its effects. Given the involvement of excitotoxicity and redox imbalance in various neurodegenerative and cognitive disorders, modulating the apelinergic and NPY signaling axes may offer a promising therapeutic strategy. Targeting these pathways could help preserve neuronal integrity, mitigate oxidative stress, and improve cognitive outcomes in conditions such as Alzheimer’s disease, Parkinson’s disease, and ischemic injury. Future studies evaluating the translational potential of Apelin‑13, optimizing dosing strategies, and exploring receptor‑specific modulators are warranted to advance these findings toward clinical application.

## Conclusıon

This study demonstrates that NPY2R is indispensable for the neuroprotective effects of Apelin‑13 against D‑glutamate–induced excitotoxicity while NPY5R contributes significantly to cognitive recovery through a more limited yet complementary role. These findings suggest a cooperative but unequal mechanism between NPY receptor subtypes in mediating the beneficial effects of Apelin‑13. Molecular and genetic based in‑vivo studies are needed to characterize the neuroprotective effects of Apelin‑13/APJ and NPY2/NPY5 receptor interactions against excitotoxic damage in a broader manner.

## Data Availability

Data will be available upon request.
